# Dynamics of *Mycobacterium* and *bovine tuberculosis* in a Human-Buffalo Population

**DOI:** 10.1155/2014/912306

**Published:** 2014-09-02

**Authors:** A. S. Hassan, S. M. Garba, A. B. Gumel, J. M.-S. Lubuma

**Affiliations:** ^1^Department of Mathematics and Applied Mathematics, University of Pretoria, Pretoria 0002, South Africa; ^2^Department of Mathematics, University of Manitoba, Winnipeg, MB, Canada R3T 2N2

## Abstract

A new model for the transmission dynamics of* Mycobacterium tuberculosis* and bovine tuberculosis in a community, consisting of humans and African buffalos, is presented. The buffalo-only component of the model exhibits the phenomenon of backward bifurcation, which arises due to the reinfection of exposed and recovered buffalos, when the associated reproduction number is less than unity. This model has a unique endemic equilibrium, which is globally asymptotically stable for a special case, when the reproduction number exceeds unity. Uncertainty and sensitivity analyses, using data relevant to the dynamics of the two diseases in the Kruger National Park, show that the distribution of the associated reproduction number is less than unity (hence, the diseases would not persist in the community). Crucial parameters that influence the dynamics of the two diseases are also identified. Both the buffalo-only and the buffalo-human model exhibit the same qualitative dynamics with respect to the local and global asymptotic stability of their respective disease-free equilibrium, as well as with respect to the backward bifurcation phenomenon. Numerical simulations of the buffalo-human model show that the cumulative number of* Mycobacterium tuberculosis* cases in humans (buffalos) decreases with increasing number of bovine tuberculosis infections in humans (buffalo).

## 1. Introduction

*Mycobacterium tuberculosis* (MTB) and bovine tuberculosis (BTB) are chronic bacterial diseases, classified amongst the closely related species that form the* M. tuberculosis* complex (MTBC) [1]. The human MTB is caused by* tubercle bacillus* (*M. tuberculosis*), while BTB is caused by* bovine bacillus* (*M. bovis*) [2]. MTB and BTB affect a wide range of hosts, including domestic livestock (such as cattle, goats, sheep, deer, and bison), wildlife (such as badgers, deer, bison, and African buffalo) which can either be reservoir or spillover, and humans [3].

MTB remains a major global health problem affecting millions of people each year [4]. It is ranked second to human immunodeficiency virus (HIV) among the leading causes of death worldwide [4]. For instance, in the year 2012, there were 8.6 million new MTB cases and 1.3 million MTB deaths globally [4]. Similarly, BTB remains a serious problem for animal and human health in many developing countries [5]. Its widespread distribution has drastic negative socioeconomic impact, affecting public health, international trade, tourism, animal mortality, and milk production [6]. For example, in Argentina, the annual loss due to BTB is estimated to be US$ 63 million [7]. A benefit/cost analyses of BTB eradication in the United States showed an actual cost of US$ 538 million between 1917 and 1992 (current programs cost approximately US$ 3.5–4.0 million* per* year [5]).

The African buffalo transmits BTB to humans,* via* aerosol or oral (as a result of consuming raw unpasteurized milk) [1]. Furthermore, BTB can be transmitted from human to human by direct contact [1]. As in cattle, the main sources of BTB transmission in buffalo are direct contact, aerosol, oral, through a bite, or contamination of a skin wound [3] (other means of transmission, such as vertical and pseudovertical [8], also occur). Similarly, MTB can be transmitted from human to human, or from human to buffalo,* via* coughing or sneezing [1]. In humans, MTB is regarded to be an airborne disease [9]. It typically affects the lungs (pulmonary TB), but can affect other parts of the body also (extrapulmonary TB) [3]. Common signs and symptoms of MTB include coughing, chest pain, fever, weakness, and weight loss. The incubation period of MTB is approximately 2 to 12 weeks. African buffalos infected with BTB show clinical signs only when the disease has reached an advanced stage (the clinical signs of BTB in buffalo at such stage include coughing, debilitation, poor body condition or emaciation, and lagging when chased by helicopter [3, 8]). The incubation period for BTB is approximately 9 months to a year, and infections can remain dormant for years (and reactivate during periods of stress or in old age) [6].

**Figure 1:**
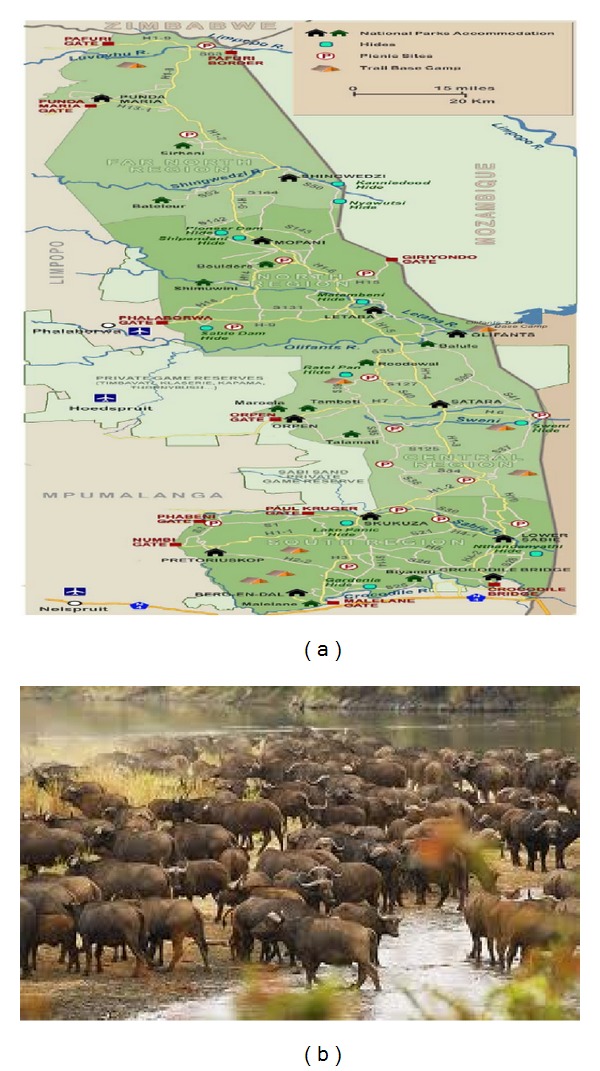
African buffalos and demographic map of Kruger National Park [11].

BTB is typically controlled using isolation or quarantine of infected herds, test-and-slaughter policy, and pasteurization of milk [10]. In South Africa's Kruger National Park (KNP), other control measures, such as culling, vaccination, and their combination, are also used [10] (a demographic map of KNP and a herd of African Buffalos [11] are shown in Figure 1). Similarly, MTB in humans is controlled* via* standard six-month course of four antimicrobial drugs [12–15]. The World Health Organization embarked on numerous global initiatives, such as “Stop TB Partnership,” “International Standards of Tuberculosis Care and Patient's Care,” and the “Global Plan to Stop TB,” with the hope of minimizing the burden of TB worldwide [12].

Several mathematical models have been developed and used to gain insight into the transmission dynamics of BTB or MTB in populations (see, for instance, [8, 10, 12, 13, 16–19] and some of the references therein). However, none of these studies incorporate humans in the transmission dynamics of BTB. The purpose of the current study is to design, and analyse, a new realistic model (which extends some of the aforementioned studies in the literature) for BTB-MTB transmission dynamics. The objective is to gain insight into the qualitative dynamics of the two diseases in a buffalo-human population.

The paper is organized as follows. The new model for BTB and MTB transmission dynamics in a community consisting of human and buffalo is presented in Section 2. The buffalo-only model is rigorously analysed in Section 3, and the full buffalo-human model is analysed in Section 4. Numerical simulations are also reported.

## 2. Model Formulation

The model to be designed is based on the transmission dynamics of MTB and BTB in a population consisting of humans and African buffalos. The total human population at time *t*, denoted by *N*_*H*_(*t*), is subdivided into seven mutually exclusive compartments of susceptible humans (*S*_*H*_(*t*)), exposed humans (who have been infected with MTB but have not yet shown clinical symptoms of the disease) (*E*_*H*1_(*t*)), exposed humans with BTB (*E*_*H*2_(*t*)), infected humans with clinical symptoms of MTB (*I*_*H*1_(*t*)), infected humans with clinical symptoms of BTB (*I*_*H*2_(*t*)), and humans who recovered from MTB (*R*_*H*1_(*t*)) or BTB (*R*_*H*2_(*t*)), so that

(1) NH(t)=SH(t)+EH1(t)+EH2(t)+IH1(t) +IH2(t)+RH1(t)+RH2(t).

Similarly, the total buffalo population (in the herd) at time *t*, denoted by *N*_*B*_(*t*), is split into susceptible (*S*_*B*_(*t*)), early-exposed with BTB (*E*_*B*1_(*t*)), early-exposed with MTB (*E*_*M*1_(*t*)), advanced-exposed with BTB (*E*_*B*2_(*t*)), advanced-exposed with MTB (*E*_*M*2_(*t*)), infected with clinical symptoms of BTB (*I*_*BB*_(*t*)), infected with clinical symptoms of MTB (*I*_*MB*_(*t*)), and recovered from BTB (*R*_*BB*_(*t*)) or MTB (*R*_*MB*_(*t*)), so that

(2) NB(t)=SB(t)+EB1(t)+EM1(t)+EB2(t) +EM2(t)+IBB(t)+IMB(t) +RBB(t)+RMB(t).

The susceptible human population (*S*_*H*_(*t*)) is increased by the recruitment of people (either by birth or immigration) into the human-buffalo poulation (at a rate Π_*H*_). The population is decreased by infection with MTB (at a rate *λ*_*H*_) or BTB (at a rate *λ*_*B*1_), where

(3) $$\begin{array}{c} \begin{array}{cc} & \lambda_{H}=\frac{\beta_{H}}{N_{H}}\left(\eta_{H1}E_{H1}+I_{H1}\right),\;\;\lambda_{B1}=\lambda_{HB}+\theta_{MM}\lambda_{B}, \end{array} \end{array}$$

with

(4) $$\begin{array}{c} \lambda_{HB}=\frac{\beta_{H}}{N_{H}}\left(\eta_{H2}E_{H2}+I_{H2}\right),\\ \lambda_{B}=\frac{\beta_{B}}{N_{B}}\left(\eta_{B1}E_{B1}+\eta_{B2}E_{B2}+I_{BB}\right). \end{array}$$

In (3) and (4), *β*_*H*_ and *β*_*B*_ represent the effective contact rates (i.e., contacts capable of leading to MTB or BTB infection), respectively. Furthermore, 0 ≤ *η*_*H*1_ < 1 and 0 ≤ *η*_*H*2_ < 1 are modification parameters accounting for the assumed reduction in infectiousness of exposed humans, in comparison to infected humans with clinical symptoms of MTB or BTB, respectively. Similarly, 0 ≤ *η*_*B*1_ < 1 and 0 ≤ *η*_*B*2_ < 1 are modification parameters accounting for the assumed reduction in infectiousness of exposed buffalos, in comparison to infected buffalos with clinical symptoms of BTB. The modification parameter 0 ≤ *θ*_*MM*_ < 1 accounts for the assumed reduced likelihood of susceptible humans acquiring BTB infection, in comparison to susceptible buffalos acquiring BTB infection. Natural death is assumed to occur in all human compartments at a rate *μ*_*H*_. Thus, the rate of change of the susceptible human population is given by

(5) $$\begin{array}{c} \frac{dS_{H}}{dt}=\Pi_{H}-\left(\lambda_{H}+\lambda_{B1}+\mu_{H}\right)S_{H}. \end{array}$$

The population of exposed humans with MTB (*E*_*H*1_(*t*)) is generated by the infection of susceptible humans with MTB (at the rate *λ*_*H*_) and is decreased by the development of clinical symptoms of MTB (at a rate *σ*_1_), exogenous reinfection (at a rate *θ*_*H*1_ *λ*_*H*_, where 0 ≤ *θ*_*H*1_ < 1 accounts for the assumption that reinfection of exposed humans with MTB occurs at a rate lower than primary infection of susceptible humans with MTB) and natural death, so that

(6) $$\begin{array}{c} \frac{dE_{H1}}{dt}=\lambda_{H}S_{H}-\left(\sigma_{1}+\theta_{H1}\lambda_{H}+\mu_{H}\right)E_{H1}. \end{array}$$

Similarly, the population of exposed humans with BTB (*E*_*H*2_(*t*)) is increased by the infection of susceptible humans with BTB (at the rate *λ*_*B*1_) and is reduced by the development of clinical symptoms of BTB (at a rate *σ*_2_), exogenous reinfection (at a rate *θ*_*H*2_ *λ*_*B*1_, with 0 ≤ *θ*_*H*2_ < 1 similarly defined as *θ*_*H*1_) and natural death. Thus,

(7) $$\begin{array}{c} \frac{dE_{H2}}{dt}=\lambda_{B1}S_{H}-\left(\sigma_{2}+\theta_{H2}\lambda_{B}+\mu_{H}\right)E_{H2}. \end{array}$$

The population of humans with clinical symptoms of MTB (*I*_*H*1_(*t*)) increases following the development of clinical symptoms of MTB by exposed humans (at the rate *σ*_1_) and exogenous reinfection of exposed and recovered humans (at the rates *θ*_*H*1_ *λ*_*H*_ and *θ*_*RH*_ *λ*_*H*_, resp., with 0 ≤ *θ*_*RH*_ < 1). This population is decreased by recovery (at a rate *γ*_1_), natural death, and MTB-induced death (at a rate *δ*_*H*1_), so that

(8) dIH1dt=σ1EH1+(θH1EH1+θRHRH1)λH −(γ1+μH+δH1)IH1.

The population of infected humans with clinical symptoms of BTB (*I*_*H*2_(*t*)) is generated by the development of clinical symptoms of BTB by exposed humans (at the rate *σ*_2_) and reinfection of exposed and recovered humans (at the rates *θ*_*H*2_ *λ*_*B*1_ and *θ*_*RB*_ *λ*_*B*1_, resp., with 0 ≤ *θ*_*RB*_ < 1). This population is decreased by recovery (at a rate *γ*_2_), natural death, and BTB-induced death (at a rate *δ*_*H*2_). This gives

(9) dIH2dt=σ2EH2+(θH2EH2+θRBRH2)λB1 −(γ2+μH+δH2)IH2.

The population of humans who recovered from MTB (*R*_*H*1_(*t*)) is generated by the recovery of humans with clinical symptoms of MTB (at the rate *γ*_1_). It is decreased by exogenous reinfection (at the rate *θ*_*RH*_ *λ*_*H*_) and natural death. Hence,

(10) $$\begin{array}{c} \frac{dR_{H1}}{dt}=\gamma_{1}I_{H1}-\left(\theta_{RH}\lambda_{H}+\mu_{H}\right)R_{H1}. \end{array}$$

It should be mentioned that, since MTB-infected humans do not completely eliminate the bacteria from their body (usually the bacteria hide in the bone marrow), “recovery” in this case implies (or represents) a long period of latency (which could last for a lifetime) [19, 30].

Similarly, the population of humans who recovered from BTB (*R*_*H*2_(*t*)) is generated by the recovery of humans with clinical symptoms of BTB (at the rate *γ*_2_) and is decreased by reinfection (at the rate *θ*_*RB*_ *λ*_*B*1_) and natural death, so that

(11) $$\begin{array}{c} \frac{dR_{H2}}{dt}=\gamma_{2}I_{H2}-\left(\theta_{RB}\lambda_{B1}+\mu_{H}\right)R_{H2}. \end{array}$$

The population of susceptible buffalos (*S*_*B*_(*t*)) is generated by the recruitment of buffalos (either by birth or restocking from other herds) at a rate Π_*B*_. It is assumed that all recruited buffalos are susceptible. The population of susceptible buffalos is decreased by acquisition of BTB infection (following effective contact with a human or buffalo infected with BTB), at the rate *λ*_*B*_ (where, *λ*_*B*_ = *θ*_*BB*_ *λ*_*HB*_ + *λ*_*BB*_, with the modification parameters 0 ≤ *θ*_*BB*_ < 1 accounting for the expected reduced likelihood of humans transmitting of BTB to buffalo, in relation to BTB transmission from a human to another human) or MTB (following effective contact with a human infected with MTB) at a reduced rate *θ*_*HH*_ *λ*_*H*_ (where 0 ≤ *θ*_*HH*_ < 1 is a modification parameter accounting for the assumed reduction in the transmissibility of MTB from humans to buffalos, in comparison to MTB transmission from humans to humans), and by natural death (at a rate *μ*_*B*_, buffalos in each epidemiological compartment suffer natural death at this rate). Thus,

(12) $$\begin{array}{c} \frac{dS_{B}}{dt}=\Pi_{B}-\left(\lambda_{B}+\theta_{HH}\lambda_{H}+\mu_{B}\right)S_{B}. \end{array}$$

An important feature of BTB transmission within the buffalo population is that an infected buffalo could be in early or advanced stage of infection. This is owing to the fact that the clinical symptoms of BTB usually take months to manifest in buffalos [6]. Thus, BTB infections can remain dormant for years and reactivate during periods of stress or in old age [6]. These (early and advanced-exposed stage) features are incorporated in the model being develop. The population of buffalos early-exposed to BTB (*E*_*B*1_(*t*)) is increased by the infection of susceptible buffalos with BTB (at the rate *λ*_*B*_). This population is decreased by exogenous reinfection with BTB (at a rate *θ*_*EB*_ *λ*_*B*_, with 0 ≤ *θ*_*EB*_ < 1), progression to the advanced-exposed class (at a rate *κ*_1_), and natural death. This gives

(13) $$\begin{array}{c} \frac{dE_{B1}}{dt}=\lambda_{B}S_{B}-\left(\theta_{EB}\lambda_{B}+\kappa_{1}+\mu_{B}\right)E_{B1}. \end{array}$$

The population of buffalos early-exposed to MTB is increased by the infection of susceptible buffalos with MTB (at the rate *θ*_*HH*_ *λ*_*H*_, where 0 ≤ *θ*_*HH*_ < 1 is as defined above). The population is decreased by exogenous reinfection (at a rate *θ*_*EB*_ *λ*_*H*_), progression to the advanced-exposed MTB class (at a rate *κ*_2_), and natural death. This gives

(14) $$\begin{array}{c} \frac{dE_{M1}}{dt}=\theta_{HH}\lambda_{H}S_{B}-\left(\theta_{EB}\lambda_{H}+\kappa_{2}+\mu_{B}\right)E_{M1}. \end{array}$$

The population of buffalos at advanced-exposed BTB class (*E*_*B*2_(*t*)) is increased by the progression of buffalos in the early-exposed BTB class (at the rate *κ*_1_). It is decreased by exogenous reinfection (at a rate *θ*_*EB*_ *λ*_*B*_), development of clinical symptoms of BTB (at a rate *σ*_*B*2_), and natural death, so that

(15) $$\begin{array}{c} \frac{dE_{B2}}{dt}=\kappa_{1}E_{B1}-\left(\theta_{EB}\lambda_{B}+\sigma_{B2}+\mu_{B}\right)E_{B2}. \end{array}$$

Similarly, the population of buffalos at advanced-exposed MTB class (*E*_*M*2_(*t*)) is generated by the progression of buffalos in the early-exposed MTB class (at the rate *κ*_2_). It is decreased by exogenous reinfection (at a rate *θ*_*EB*_ *λ*_*H*_), development of clinical symptoms of MTB (at a rate *σ*_*M*2_), and natural death. Hence,

(16) $$\begin{array}{c} \frac{dE_{M2}}{dt}=\kappa_{2}E_{M1}-\left(\theta_{EB}\lambda_{H}+\sigma_{M2}+\mu_{B}\right)E_{M2}. \end{array}$$

The population of buffalos with clinical symptoms of BTB (*I*_*BB*_(*t*)) is increased by the development of clinical symptoms of exposed buffalos with BTB (at the rate *σ*_*B*2_) and by the exogenous reinfection of exposed and recovered buffalos (at the rates *θ*_*EB*_ *λ*_*B*_ and *θ*_*RB*_ *λ*_*B*_, resp.). It is decreased by recovery (at a rate *γ*_*B*1_), natural death, and BTB-induced mortality (at a rate *δ*_*B*_). Thus,

(17) dIBBdt=σB2EB2+(EB1+EB2)θEBλB+θRBλBRBB −(γB1+μB+δB)IBB.

The population of buffalos with clinical symptoms of MTB (*I*_*MB*_(*t*)) is increased by the development of clinical symptoms of exposed buffalos with MTB (at the rate *σ*_*M*2_) and by the exogenous reinfection of exposed and recovered buffalos (at the rates *θ*_*EB*_ *λ*_*H*_ and *θ*_*RB*_ *λ*_*H*_, resp.). It is decreased by recovery (at a rate *γ*_*M*1_), natural death, and MTB-induced mortality (at a rate *δ*_*M*_). Thus,

(18) dIMBdt=σM2EM2+(EM1+EM2)θEBλH+θRBλHRMB −(γM1+μB+δM)IMB.

The population of buffalos who recovered from BTB (*R*_*BB*_(*t*)) is increased following the recovery of buffalos with clinical symptoms of BTB (at the rate *γ*_*B*1_). It is decreased by reinfection (at the rate *θ*_*RB*_ *λ*_*B*_) and natural death, so that

(19) $$\begin{array}{c} \frac{dR_{BB}}{dt}=\gamma_{B1}I_{BB}-\left(\theta_{RB}\lambda_{B}+\mu_{B}\right)R_{BB}. \end{array}$$

Finally, the population of buffalos who recovered from MTB (*R*_*MB*_(*t*)) is generated by the recovery of buffalos with MTB (at the rate *γ*_*M*1_) and is decreased following reinfection (at the rate *θ*_*RB*_ *λ*_*H*_) and natural death. This gives

(20) $$\begin{array}{c} \frac{dR_{MB}}{dt}=\gamma_{\text{M}1}I_{MB}-\left(\theta_{RB}\lambda_{H}+\mu_{B}\right)R_{MB}. \end{array}$$

It is assumed that recovered buffalos and humans acquire permanent natural immunity against BTB or MTB infection so that recovered buffalos and humans do not return to their respective susceptible class (*albeit* buffalos and humans in recovered classes can acquire reinfection).

**Figure 2:**
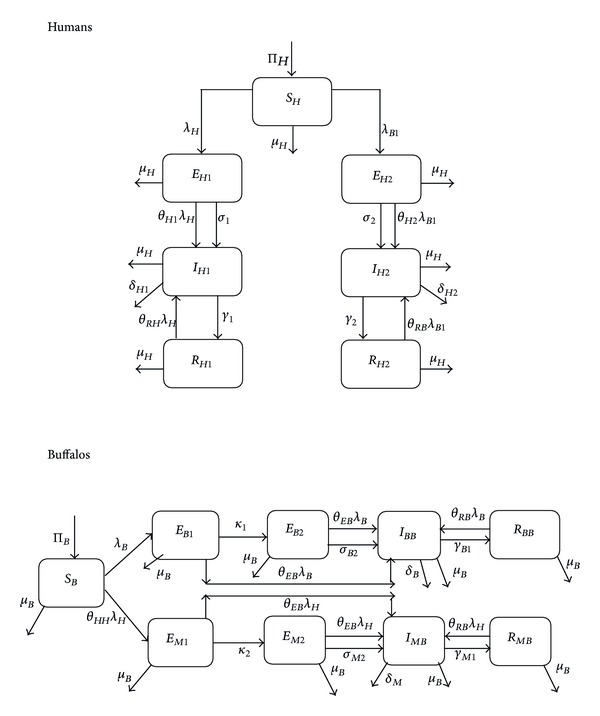
Schematic diagram of the BTB-MTB model (21).

Thus, based on the above assumptions and formulations, the model for the BTB-MTB transmission dynamics in a human-buffalo population is given by the following deterministic system of nonlinear differential equations (a flow diagram of the model is depicted in Figure 2, and the associated variables and parameters are described in Tables 1 and 2, resp.):

(21) Human  ComponentdSHdt=ΠH−(λH+λB1+μH)SH,dEH1dt=λHSH−(θH1λH+σ1+μH)EH1,dEH2dt=λB1SH−(θH2λB1+σ2+μH)EH2,dIH1dt=σ1EH1+(θH1EH1+θRHRH1)λH−(γ1+μH+δH1)IH1,dIH2dt=σ2EH2+(θH2EH2+θRBRH2)λB1−(γ2+μH+δH2)IH2,dRH1dt=γ1IH1−(θRHλH+μH)RH1,dRH2dt=γ2IH2−(θRBλB1+μH)RH2.Buffalo  ComponentdSBdt=ΠB−(θHHλH+λB+μB)SB,dEB1dt=λBSB−(θEBλB+κ1+μB)EB1,dEM1dt=θHHλHSB−(θEBλH+κ2+μB)EM1,dEB2dt=κ1EB1−(θEBλB+σB2+μB)EB2,dEM2dt=κ2EM1−(θEBλH+σM2+μB)EM2,dIBBdt=σB2EB2+(EB1+EB2)θEBλB+θRBλBRBB−(γB1+μB+δB)IBB,dIMBdt=σM2EM2+(EM1+EM2)θEBλH+θRBλHRMB−(γM1+μB+δM)IMB,dRBBdt=γB1IBB−(θRBλB+μB)RBB,dRMBdt=γM1IMB−(θRBλH+μB)RMB.

The model (21) is, to the authors' knowledge, the first to incorporate humans and MTB dynamics in the transmission dynamics of BTB in a human-buffalo community. Furthermore, it extends numerous models for BTB transmission in the literature, such as those in [8, 12, 13, 16–19], by,* inter alia*,

- including the dynamics of early and advanced-exposed buffalos (exposed buffalo classes were not considered in the models in [8, 12, 16–18]),
- allowing for BTB and MTB transmission by exposed buffalos and humans (this was not considered in [8, 12, 16–19]),
- including the dynamics of humans (this was not considered in [8, 13, 18, 19]),
- allowing for the reinfection of exposed and recovered buffalos and humans (this was not considered in [8, 12, 13, 16, 18]),
- allowing for the transmission of both BTB and MTB in both the buffalo and human populations (this was not considered in the models in [8, 12, 13, 16–18]).

The model (21) will now be rigorously analysed to gain insight into its dynamical features. Before doing so, it is instructive, however, to consider the dynamics within the buffalo population only as below.

## 3. Analysis of Buffalo-Only Model

Consider the model (21) in the absence of humans (buffalo-only model), obtained by setting the human components to zero (i.e., setting *S*_*H*_ = *E*_*H*1_ = *E*_*H*2_ = *I*_*H*1_ = *I*_*H*2_ = *R*_*H*1_ = *R*_*H*2_ = *λ*_*H*_ = *θ*_*HH*_ = 0 in (21)), given by

(22) dSBdt=ΠB−(λB+μB)SB,dEB1dt=λBSB−(θEBλB+κ1+μB)EB1,dEM1dt=−(κ2+μB)EM1,dEB2dt=κ1EB1−(θEBλB+σB2+μB)EB2,dEM2dt=κ2EM1−(σM2+μB)EM2,dIBBdt=σB2EB2+(EB1+EB2)θEBλB+θRBλBRBB−(γB1+μB+δB)IBB,dIMBdt=σM2EM2−(γM1+μB+δM)IMB,dRBBdt=γB1IBB−(θRBλB+μB)RBB,dRMBdt=γM1IMB−μBRMB,

where, now,

(23) $$\begin{array}{c} \lambda_{B}=\frac{\beta_{B}}{N_{B}}\left(\eta_{B1}E_{B1}+\eta_{B2}E_{B2}+I_{BB}\right). \end{array}$$

**Figure 3:**
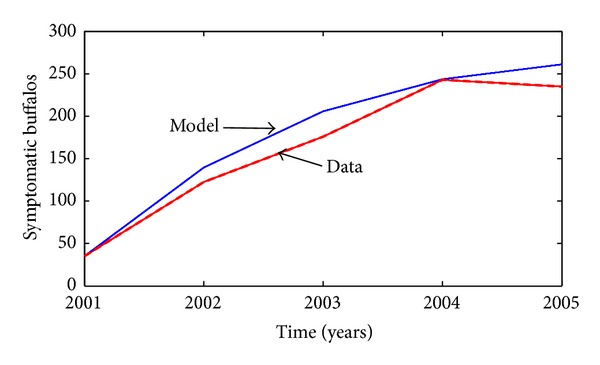
Data fit of the simulation of the buffalo-only model (22), using data obtained from South Africa's Kruger National Park (Table 4) [29]. Parameter values used are as given in Table 3.

The buffalo-only model (22) is fitted using data obtained from South Africa's Kruger National Park [29], as shown in Figure 3 (from which it is evident that the model mimics the data reasonably well).

It is worth stating that since there are no humans in the dynamics of the buffalo-only model (22), MTB is not transmitted to susceptible buffalos. Furthermore, it is clear from the third equation in (22) that

(24) $$\begin{array}{c} E_{M1}\left(t\right)⟶0\;\text{as}\;t⟶\infty . \end{array}$$

Substituting (24) in the fifth equation in (22) shows that

(25) $$\begin{array}{c} E_{M2}\left(t\right)⟶0\;\text{as}\;t⟶\infty . \end{array}$$

Similarly, by substituting (*E*_*M*1_, *E*_*M*2_) = (0,0) into the equations for *I*_*MB*_ and *R*_*MB*_ in (22), it follows that

(26) $$\begin{array}{c} \left(I_{MB}\left(t\right),R_{MB}\left(t\right)\right)⟶\left(0,0\right)\;\text{as}\;t⟶\infty . \end{array}$$

Thus, the buffalo-only model reduces to the following (limited) model at steady-state:

(27) dSBdt=ΠB−(λB+μB)SB,dEB1dt=λBSB−(θEBλB+κ1+μB)EB1,dEB2dt=κ1EB1−(θEBλB+σB2+μB)EB2,dIBBdt=σB2EB2+(EB1+EB2)θEBλB+θRBλBRBB−(γB1+μB+δB)IBB,dRBBdt=γB1IBB−(θRBλB+μB)RBB.

**Lemma 1 .**

The following biologically feasible region of the buffalo-only model (27)

(28) Γ={(SB,EB1,EB2,IBB,RBB)∈R+5:SB+EB1 +EB2+IBB+RBB≤ΠBμB}

is positively invariant and attracting.

**Proof**

Adding the equations in the buffalo-only model system (27) gives

(29) $$\begin{array}{c} \frac{dN_{B}\left(t\right)}{dt}=\Pi_{\text{B}}-\mu_{B}N_{B}\left(t\right)-\delta_{B}I_{BB}\left(t\right), \end{array}$$

so that

(30) $$\begin{array}{c} \frac{dN_{B}\left(t\right)}{dt}\leq \Pi_{B}-\mu_{B}N_{B}\left(t\right). \end{array}$$

It follows from (30) and the Gronwall inequality that

(31) $$\begin{array}{c} N_{B}\left(t\right)\leq N_{B}\left(0\right){\text{e}}^{-\mu_{B}(t)}+\frac{\Pi_{B}}{\mu_{B}}\left[1-{\text{e}}^{-\mu_{B}(t)}\right]. \end{array}$$

In particular, *N*_*B*_(*t*) ≤ Π_*B*_/*μ*_*B*_ if *N*_*B*_(0) ≤ Π_*B*_/*μ*_*B*_. Thus, Γ is positively invariant. Hence, it is sufficient to consider the dynamics of the buffalo-only model (27) in Γ (where the model can be considered to be epidemiologically and mathematically well-posed [31]).

**Theorem 2 .**

Let the initial data *S*_*B*_(0) > 0, *E*_*B*1_(0) > 0, *E*_*B*2_(0) > 0, *I*_*BB*_(0) > 0, *R*_*BB*_(0) > 0. Then, the solutions *S*_*B*_(*t*), *E*_*B*1_(*t*), *E*_*B*2_(*t*), *I*_*BB*_(*t*), and *R*_*BB*_(*t*) of the buffalo-only model (27) are positive for all *t* ≥ 0.

**Proof**

It is clear from the first equation of the buffalo-only model (27) that

(32) $$\begin{array}{c} \frac{dS_{B}}{dt}\geq -\left(\lambda_{B}+\mu_{B}\right)S_{B}, \end{array}$$

so that

(33) $$\begin{array}{c} S_{B}\left(t\right)\geq S_{B}\left(0\right)\exp\left[-\overset{t}{\underset{0}{\int }}\left(\lambda_{B}+\mu_{B}\right)du\right]>0,\;\forall t>0. \end{array}$$

Using similar approach, it can be shown that *E*_*B*1_(*t*) > 0, *E*_*B*2_(*t*) > 0, *I*_*BB*_(*t*) > 0, and *R*_*BB*_(*t*) > 0, for all *t* > 0.

### 3.1. Asymptotic Stability of Disease-Free Equilibrium (DFE)

#### 3.1.1. Local Asymptotic Stability

The DFE of the buffalo-only model (27) is given by

(34) $$\begin{array}{c} E_{0}=\left(S_{B}^{*},E_{B1}^{*},E_{B2}^{*},I_{BB}^{*},R_{BB}^{*}\right)=\left(\frac{\Pi_{B}}{\mu_{B}},0,0,0,0\right). \end{array}$$

The linear stability of *E*_0_ can be established using the next generation operator method on the system (22) [32, 33]. The matrices *F* (for the new infection terms) and *V* (of the transition terms) associated with the system (27) are given, respectively, by

(35) F=[βBηB1βBηB2βB000000],  V=[K100−κ1K300−σB2K5],

where *K*_1_ = *κ*_1_ + *μ*_*B*_, *K*_3_ = *σ*_*B*2_ + *μ*_*B*_, and *K*_5_ = *γ*_*B*1_ + *μ*_*B*_ + *δ*_*B*_. It follows that the* basic reproduction number* of the buffalo-only model (27), denoted by *R*_0_, is given by

(36) $$\begin{array}{c} R_{0}=\frac{\beta_{B}\left[\eta_{B1}K_{3}K_{5}+\kappa_{1}\left(\eta_{B2}K_{5}+\sigma_{B2}\right)\right]}{K_{1}K_{3}K_{5}}. \end{array}$$

Hence, using Theorem 2 of [33], the following result is established.

**Lemma 3 .**

The DFE, *E*_0_, of the buffalo-only model (27), is locally asymptotically stable (LAS) if *R*_0_ < 1 and unstable if *R*_0_ > 1.

The threshold quantity, *R*_0_, represents the average number of secondary cases of BTB in the buffalo population that one BTB-infected buffalo can generate if introduced into a completely susceptible buffalo population [31, 34, 35].

#### 3.1.2. Interpretation of *R*_0_

The threshold quantity, *R*_0_, can be interpreted as follows. It is worth recalling, first of all, that susceptible buffalos can acquire BTB infection following effective contact with either early-exposed buffalo with BTB (*E*_*B*1_(*t*)), advanced-exposed buffalo with BTB (*E*_*B*2_(*t*)), or infected buffalo with clinical symptoms of BTB (*I*_*BB*_(*t*)). It follows that the number of BTB infections generated by an early-exposed buffalo (near the DFE) is given by the product of the infection rate of an early-exposed buffalo (*β*_*B*_ *η*_*B*1_/*N*_B_*) and the average duration of stay in the early-exposed class (1/*K*_1_). Thus, the average number of BTB infections generated by early-exposed buffalos is given by

(37) $$\begin{array}{c} \frac{\beta_{B}\eta_{B1}S_{B}^{*}}{K_{1}N_{B}^{*}}. \end{array}$$

Similarly, the number of BTB infections generated by an advanced-exposed buffalo (near the DFE) is given by the product of the infection rate of advanced-exposed buffalos (*β*_*B*_ *η*_*B*2_/*N*_B_*), the probability that early-exposed buffalo survived the early-exposed class and move to the advanced-exposed class (*κ*_1_/*K*_1_), and the average duration of stay in the advanced-exposed class (1/*K*_3_). Thus, the average number of BTB infections generated by advanced-exposed buffalos is given by

(38) $$\begin{array}{c} \frac{\beta_{B}\eta_{B2}\kappa_{1}S_{B}^{*}}{K_{1}K_{3}N_{B}^{*}}. \end{array}$$

Furthermore, the number of BTB infections generated by an infected buffalo with clinical symptoms of BTB (near the DFE) is given by the product of the infection rate of buffalos with clinical symptoms of BTB (*β*_*B*_/*N*_B_*), the probability that an advanced-exposed buffalo survived the advanced-exposed class and move to the symptomatic class *I*_*BB*_ (*κ*_1_ *σ*_*B*2_/*K*_1_ *K*_3_), and the average duration of stay in the symptomatic class *I*_*BB*_ (1/*K*_5_). Thus, the average number of BTB infections generated by advanced-exposed buffalos is given by

(39) $$\begin{array}{c} \frac{\beta_{B}\kappa_{1}\sigma_{B2}S_{B}^{*}}{K_{1}K_{3}K_{5}N_{B}^{*}}. \end{array}$$

The sum of the terms in (37), (38), and (39) gives *R*_0_. That is, the average number of new infections generated by infected buffalos (early-exposed, advanced-exposed, or symptomatic) is given by (noting that *S*_B_* = Π_*B*_/*μ*_*B*_ and *N*_B_* = Π_*B*_/*μ*_*B*_)

(40) $$\begin{array}{c} R_{0}=\frac{\beta_{B}\left[\eta_{B1}K_{3}K_{5}+\kappa_{1}\left(\eta_{B2}K_{5}+\sigma_{B2}\right)\right]}{K_{1}K_{3}K_{5}}. \end{array}$$

The epidemiological implication of Lemma 3 is that BTB can be effectively controlled in (or eliminated from) the buffalo population (herd) if the initial sizes of the state variables of the buffalo-only model (27) are in the basin of attraction of the DFE (*E*_0_). It is worth mentioning, however, that TB models with exogenous reinfection are often shown to exhibit the phenomenon of backward bifurcation (where the stable DFE coexists with a stable endemic equilibrium when *R*_0_ < 1 [12, 17, 25, 36]). The epidemiological implication of this phenomenon is that the classical requirement of *R*_0_ < 1 is, although necessary, no longer sufficient for diseases elimination [12, 36]. Thus, the presence of backward bifurcation in the transmission dynamics of a disease makes its effective control in a population more difficult. Hence, it is instructive to explore the possibility of such phenomenon in the buffalo-only model (22). This is investigated below.

**Theorem 4 .**

The buffalo-only model (22) undergoes backward bifurcation at *R*_0_ = 1 whenever the bifurcation coefficient, *a*, given by (A.9) (in Appendix A), is positive.

The proof of Theorem 4, based on using centre manifold theory [17, 33], is given in Appendix A. It should be noted that, in the absence of reinfection of exposed and recovered buffalos (i.e., the case of the model (22) with *θ*_*EB*_ = *θ*_*RB*_ = 0), the backward bifurcation coefficient, *a*, given by (A.9) in Appendix A, reduces to (it should be noted, from Appendix A, that *A*_1_ > 0, *A*_2_ > 0, *β*_B_* > 0, and, from Theorem 2, that all parameters of the buffalo-only model (22) are nonnegative):

(41) −2βB∗μB2ΠB{1+μB(K5+σB1)+σB2γB1A1μBσB2+γM1σM2K2+K2μBK6+σB2A2K2μBσM2},<0,

where *K*_2_ = *κ*_2_ + *μ*_*B*_, *K*_4_ = *σ*_*M*2_ + *μ*_*B*_ and *K*_6_ = *γ*_*M*1_ + *μ*_*B*_ + *δ*_*M*_. Since the bifurcation coefficient, *a*, is automatically negative, it follows from the analyses in Appendix A, and Theorem 4.1 of [17], that the buffalo-only model (22) does not undergo backward bifurcation in the absence of reinfection (this result is consistent with that in [12, 17, 36], on the transmission dynamics* Mycobacterium tuberculosis* in human populations). This result is summarized below.

**Lemma 5 .**

The buffalo-only model (22) does not undergo backward bifurcation at *R*_0_ = 1 in the absence of the reinfection of exposed and recovered buffalos (*θ*_*EB*_ = *θ*_*RB*_ = 0).

Hence, this study shows that the reinfection of exposed and recovered buffalos causes the phenomenon of backward bifurcation in the transmission dynamics of BTB and MTB in a buffalo-only population. To further confirm the absence of backward bifurcation in this case, a global asymptotic stability result is established for the DFE (*E*_0_) of the buffalo-only model (27) in the absence of reinfection (i.e., *θ*_*EB*_ = *θ*_*RB*_ = 0) below.

### 3.2. Global Asymptotic Stability of the DFE

Consider the buffalo-only model (27) in the absence of the reinfection of exposed (*θ*_*EB*_ = 0) and recovered (*θ*_*RB*_ = 0) buffalos.

**Theorem 6 .**

The DFE, *E*_0_, of the buffalo-only model (27) with *θ*_*EB*_ = *θ*_*RB*_ = 0 is globally asymptotically stable (GAS) in Γ if *R*_0_ ≤ 1.

**Proof**

Consider the buffalo-only model (27) in the absence of reinfection (*θ*_*EB*_ = *θ*_*RB*_ = 0). Furthermore, let *R*_0_ ≤ 1. Consider the linear Lyapunov function *F* = *a*_0_ *E*_*B*1_ + *a*_1_ *E*_*B*2_ + *a*_2_ *I*_*BB*_, where

(42) $$\begin{array}{c} a_{0}=R_{0},\;\;a_{1}=\frac{\beta_{B}\left(\eta_{B2}K_{5}+\sigma_{B2}\right)}{K_{3}K_{5}},\;\;a_{2}=\frac{\beta_{B}}{K_{5}}, \end{array}$$

with Lyapunov derivative given by (where a dot represents differentiation with respect to time *t*)

(43) F˙=a0E˙B1+a1E˙B2+a2I˙BB,=a0[βBNB(ηB1EB1+ηB2EB2+IBB)SB−K1EB1] +a1(κ1EB1−K3EB2)+a2(σB2EB2−K5IBB),=(a0βBηB1SBNB−a0K1+a1κ1)EB1 +(a0βBηB2SBNB−a1K3+a2σB2)EB2 +(a0βBSBNB−a2K5)IBB,≤βB(ηB1EB1+ηB2EB2+IBB)(R0−1)since  SB(t)≤NB(t) ∀t  in  Γ,≤0 if  R0≤1.

Since all the parameters and variables of model (27) are nonnegative (Theorem 2), it follows that $\dot{F}\leq 0$ for *R*_0_ ≤ 1 with $\dot{F}=0$ if and only if *E*_*B*1_ = *E*_*B*2_ = *I*_*BB*_ = 0. Thus, it follows, by LaSalle's Invariance Principle [37], that

(44) $$\begin{array}{c} \left(E_{B1}\left(t\right),E_{B2}\left(t\right),I_{BB}\left(t\right)\right)⟶\left(0,0,0\right)\;\text{as}\;t⟶\infty . \end{array}$$

Since lim⁡_*t*→*∞*_⁡sup⁡*I*_*BB*_(*t*) = 0 (from (44)), it follows that, for sufficiently small ϖ* > 0, there exists a constant *M* > 0, such that lim⁡_*t*→*∞*_⁡sup⁡*I*_*BB*_(*t*) ≤ ϖ* for all *t* > *M*. Hence, it follows from the fifth equation of the buffalo-only model (27) that, for $t>M,{\dot{R}}_{BB}\leq \gamma_{B1}\varpi^{*}-\mu_{B}R_{BB}$. Thus, by comparison theorem [38], *R*_*BB*_^*∞*^ = lim⁡_*t*→*∞*_⁡sup⁡*R*_*BB*_ ≤ *γ*_*B*1_ ϖ*/*μ*_*B*_, so that, by letting, ϖ* → 0,

(45) $$\begin{array}{c} R_{BB}^{\infty }=\underset{t\to \infty }{\lim}\;\sup R_{BB}\left(t\right)\leq 0. \end{array}$$

Similarly, it can be shown that

(46) $$\begin{array}{c} {R_{BB}}_{\infty }=\underset{t\to \infty }{\lim}\;\inf R_{BB}\left(t\right)\geq 0. \end{array}$$

Thus, it follows from (45) and (46), that *R*_*BB*__*∞*_ ≥ 0 ≥ *R*_*BB*_^*∞*^. Hence,

(47) $$\begin{array}{c} \underset{t\to \infty }{\lim}R_{BB}\left(t\right)=0. \end{array}$$

Furthermore, substituting (44) in the first equation of (27) shows that

(48) $$\begin{array}{c} S_{B}\left(t\right)⟶\frac{\Pi_{B}}{\mu_{B}}\;\text{as}\;t⟶\infty . \end{array}$$

Thus, by combining equations (44), (47), and (48), it follows that every solution of the equations of the buffalo-only model (27), with *θ*_*EB*_ = *θ*_*RB*_ = 0 and initial conditions in Γ, approaches *E*_0_ as *t* → *∞* (whenever *R*_0_ ≤ 1).

**Figure 4:**
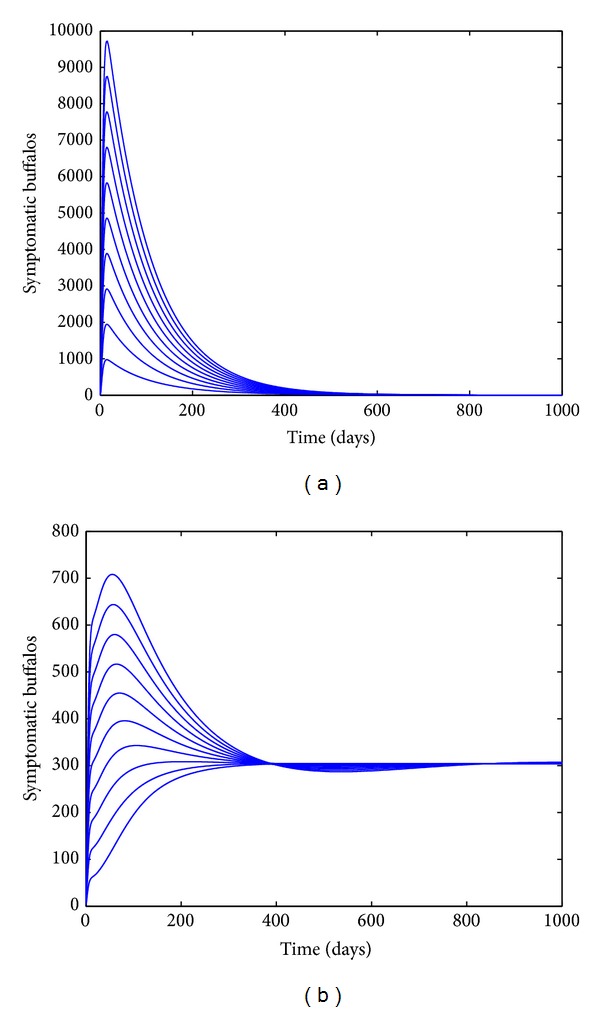
Simulations of the buffalo-only model (27), showing the total number of infected buffalos with clinical symptoms of BTB (*I*_*BB*_(*t*)(*t*)) at time *t* as a function of time. Parameter values used are as given in Table 3 with (a) *β*_*B*_ = 0.00733 (so that, *R*_0_ = 0.7036 < 1) and (b) *β*_*B*_= 0.0733, *δ*_*B*_ = 0 (so that, ${\tilde{R}}_{0}=8.6050>1$).

Theorem 6 shows that, in the absence of the reinfection of exposed and recovered buffalos (i.e., *θ*_*EB*_ = *θ*_*RB*_ = 0), BTB can be eliminated from the buffalo-only population if the reproduction number of the model (*R*_0_) can be brought to (and maintained at) a value less than unity.  Figure 4(a) depicts the solution profiles of the buffalo-only model (27), generated using various initial conditions, showing convergence to the DFE *E*_0_ when *R*_0_ < 1 (in line with Theorem 6).

### 3.3. Existence of Endemic Equilibria: Special Case

In this section, the existence of nontrivial (endemic) equilibria (where the components of the infected variables of the model are nonzero) of the buffalo-only model (27) is explored for the special case without reinfection (i.e., *θ*_*EB*_ = *θ*_*RB*_ = 0). Solving the equations of the buffalo-only model (27) at steady-state gives the following general form of the endemic equilibrium (denoted by *E*_1_):

(49) $$\begin{array}{c} E_{1}=\left(S_{B}^{**},E_{B1}^{**},E_{B2}^{**},I_{BB}^{**},R_{BB}^{**}\right), \end{array}$$

where

(50) $$\begin{array}{c} S_{B}^{**}=\frac{\Pi_{B}}{\lambda_{B}^{**}+\mu_{B}},\;\;E_{B1}^{**}=\frac{\lambda_{B}^{**}\Pi_{B}}{K_{1}\left(\lambda_{B}^{**}+\mu_{B}\right)},\\ E_{B2}^{**}=\frac{\kappa_{1}\lambda_{B}^{**}\Pi_{B}}{K_{1}K_{3}\left(\lambda_{B}^{**}+\mu_{B}\right)},\;\;I_{BB}^{**}=\frac{\sigma_{B2}\kappa_{1}\lambda_{B}^{**}\Pi_{B}}{K_{1}K_{3}K_{5}\left(\lambda_{B}^{**}+\mu_{B}\right)},\\ R_{BB}^{**}=\frac{\gamma_{B1}\sigma_{B2}\kappa_{1}\lambda_{B}^{**}\Pi_{B}}{K_{1}K_{3}K_{5}\mu_{B}\left(\lambda_{B}^{**}+\mu_{B}\right)}, \end{array}$$

with the force of infection at steady-state (*λ*_B_**) given by

(51) $$\begin{array}{c} \lambda_{B}^{**}=\frac{\beta_{B}}{N_{B}^{**}}\left(\eta_{B1}E_{B1}^{**}+\eta_{B2}E_{B2}^{**}+I_{BB}^{**}\right). \end{array}$$

Using (50) in the expression for *λ*_B_** in (51) shows that the nonzero equilibrium of the model (22) satisfies the linear equation:

(52) $$\begin{array}{c} b_{1}\lambda_{B}^{**}+b_{2}=0, \end{array}$$

where *b*_1_ = *K*_5_ *μ*_*B*_(*K*_3_ + *κ*_1_) + *σ*_*B*2_ *κ*_1_(*μ*_*B*_ + *γ*_*B*2_) and *b*_2_ = *K*_1_ *K*_3_ *K*_5_ *μ*_*B*_(1 − *R*_0_). Clearly, the coefficient *b*_1_ is always positive, and *b*_2_ is positive (negative) if *R*_0_ is less than (greater than) unity, respectively. Thus, the linear system (52) has a unique positive solution, given by *λ*_B_** = −*b*_2_/*b*_1_, whenever *R*_0_ > 1. Further, the force of infection for buffalos (*λ*_B_**) is negative whenever *R*_0_ < 1 (which is biologically meaningless). Hence, the buffalo-only model (27) has no positive equilibrium in this case. These results are summarized below.

**Theorem 7 .**

The buffalo-only model (27), with *θ*_*EB*_ = *θ*_*RB*_ = 0, has a unique endemic equilibrium, *E*_1_, whenever *R*_0_ > 1, and no endemic equilibrium otherwise.

#### 3.3.1. Global Asymptotic Stability of Endemic Equilibrium

The global asymptotic stability of the unique endemic equilibrium (*E*_1_) of the buffalo-only model is explored for the special case without reinfection (*θ*_*EB*_ = *θ*_*RB*_ = 0) and BTB-induced death in buffalos (*δ*_*B*_ = 0). It is convenient to define

(53) Γ1={(SB,EB1,EB2,IBB,RBB)∈Γ:EB1=EB2=IBB=RBB=0},

the stable manifold of the DFE (*E*_0_) of the buffalo-only model (27).

**Theorem 8 .**

The unique endemic equilibrium (*E*_1_) of the buffalo-only model (27), with *θ*_*EB*_ = *θ*_*RB*_ = *δ*_*B*_ = 0, is GAS in Γ∖Γ_1_ if ${\tilde{R}}_{0}={R_{0}|}_{\delta_{B}=0}>1$.

**Proof**

Consider the buffalo-only model (27) with *θ*_*EB*_ = *θ*_*RB*_ = *δ*_*B*_ = 0. For this case, it follows from Theorem 7 that the buffalo-only model (27) has a unique endemic equilibrium whenever ${\tilde{R}}_{0}>1$. Furthermore, setting *δ*_*B*_ = 0 in model (27) shows that *N*_*B*_(*t*) → Π_*B*_/*μ*_*B*_ as *t* → *∞*. Consider the following nonlinear Lyapunov function (of Goh-Volterra type) for the subsystem of model (27) involving the state variables *S*_*B*_, *E*_*B*1_, *E*_*B*2_, and *I*_*BB*_ (noting that *N*_*B*_(*t*) is now replaced by its limiting value Π_*B*_/*μ*_*B*_):

(54) F=SB−SB∗∗−SB∗∗ln⁡(SBSB∗∗) +EB1−EB1∗∗−EB1∗∗ln⁡(EB1EB1∗∗) +(β~BηB2SB∗∗EB2∗∗+β~BSB∗∗IBB∗∗κ1EB1∗∗) ×[EB2−EB2∗∗−EB2∗∗ln⁡(EB2EB2∗∗)] +β~BSB∗∗IBB∗∗σB2EB2∗∗[IBB−IBB∗∗−IBB∗∗ln⁡(IBBIBB∗∗)],

where ${\tilde{\beta }}_{B}=\mu_{B}\beta_{B}/\Pi_{B}$. The Lyapunov derivative of *F* is given by

(55) F˙=S˙B−SB∗∗SBS˙B+E˙B1−EB1∗∗EB1E˙B1 +(β~BηB2SB∗∗EB2∗∗+β~BSB∗∗IBB∗∗κ1EB1∗∗)(E˙B2−EB2∗∗EB2E˙B2) +β~BSB∗∗IBB∗∗σB2EB2∗∗(I˙BB−IBB∗∗IBBI˙BB),=ΠB−β~B(ηB1EB1+ηB2EB2+IBB)SB−μBSB −SB∗∗SB[ΠB−β~B(ηB1EB1+ηB2EB2+IBB)SB−μBSB] +β~B(ηB1EB1+ηB2EB2+IBB)SB−K1EB1 −EB1∗∗EB1[β~B(ηB1EB1+ηB2EB2+IBB)SB−K1EB1] +(β~BηB2SB∗∗EB2∗∗+β~BSB∗∗IBB∗∗κ1EB1∗∗) ×[κ1EB1−K3EB2−EB2∗∗EB2(κ1EB1−K3EB2)] +β~BSB∗∗IBB∗∗σB2EB2∗∗[σB2EB2−K5IBB−IBB∗∗IBB ×(σB2EB2−K5IBB)].

Using the following steady-state relations (obtained from (27)),

(56) $$\begin{array}{c} \Pi_{B}={\tilde{\beta }}_{B}\left(\eta_{B1}E_{B1}^{**}+\eta_{B2}E_{B2}^{**}+I_{BB}^{**}\right)S_{B}^{**}+\mu_{B}S_{B}^{**},\\ \kappa_{1}E_{B1}^{**}=K_{3}E_{B2}^{**},\\ {\tilde{\beta }}_{B}\left(\eta_{B1}E_{B1}^{**}+\eta_{B2}E_{B2}^{**}+I_{BB}^{**}\right)S_{B}^{**}=K_{1}E_{B1}^{**},\\ \sigma_{B2}E_{B2}^{**}=K_{5}I_{BB}^{**},\;\;\gamma_{B1}I_{BB}^{**}=\mu_{B}R_{BB}^{**}, \end{array}$$

the Lyapunov derivative can be simplified to

(57) F˙=β~B(ηB1EB1∗∗+ηB2EB2∗∗+IBB∗∗)SB∗∗ +μBSB∗∗−μBSB −SB∗∗SB[β~B(ηB1EB1∗∗+ηB2EB2∗∗+IBB∗∗)SB∗∗+μBSB∗∗−  β~B(ηB1EB1+ηB2EB2+IBB)SB−μBSB] −K1EB1−EB1∗∗EB1[β~B(ηB1EB1+ηB2EB2+IBB)SB−  K1EB1] +(β~BηB2SB∗∗EB2∗∗+β~BSB∗∗IBB∗∗κ1EB1∗∗) ×[κ1EB1−K3EB2−EB2∗∗EB2(κ1EB1−K3EB2)] +β~BSB∗∗IBB∗∗σB2EB2∗∗ ×[σB2EB2−K5IBB−IBB∗∗IBB(σB2EB2−K5IBB)].

Thus,

(58) F˙=μBSB∗∗(2−SB∗∗SB−SBSB∗∗) +β~BηB1EB1∗∗SB∗∗(2−SB∗∗SB−SBSB∗∗) +β~BηB2EB2∗∗SB∗∗(3−SB∗∗SB−EB1EB2∗∗EB1∗∗EB2−SBEB1∗∗EB2SB∗∗EB1EB2∗∗) +β~BIBB∗∗SB∗∗ ×(4−SB∗∗SB−EB1EB2∗∗EB1∗∗EB2−EB2IBB∗∗EB2∗∗IBB−SBEB1∗∗IBBSB∗∗EB1IBB∗∗).

Finally, since the arithmetic mean exceeds the geometric mean, it follows then that

(59) μBSB∗∗(2−SB∗∗SB−SBSB∗∗)≤0,β~BηB1EB1∗∗SB∗∗(2−SB∗∗SB−SBSB∗∗)≤0,β~BηB2EB2∗∗SB∗∗(3−SB∗∗SB−EB1EB2∗∗EB1∗∗EB2−SBEB1∗∗EB2SB∗∗EB1EB2∗∗) ≤0,β~BIBB∗∗SB∗∗(4−SB∗∗SB−EB1EB2∗∗EB1∗∗EB2−EB2IBB∗∗EB2∗∗IBB−SBEB1∗∗IBBSB∗∗EB1IBB∗∗) ≤0.

Furthermore, since all the model parameters are nonnegative, it follows that $\dot{F}\leq 0$ for ${\tilde{R}}_{0}>1$. Thus, $\dot{F}$ is a Lyapunov function for the subsystem of model (27) on Γ∖Γ_1_. Therefore, it follows, by LaSalle's Invariance Principle [37], that

(60) $$\begin{array}{c} \underset{t\to \infty }{\lim}S_{B}\left(t\right)=S_{B}^{**},\;\;\underset{t\to \infty }{\lim}E_{B1}\left(t\right)=E_{B1}^{**},\\ \underset{t\to \infty }{\lim}E_{B2}\left(t\right)=E_{B2}^{**},\;\;\underset{t\to \infty }{\lim}I_{BB}\left(t\right)=I_{BB}^{**}. \end{array}$$

Since *I*_*BB*_(*t*) → *I*_BB_** as *t* → *∞*, it follows from the equation for *dR*_*BB*_/*dt* in (27) that *R*_*BB*_(*t*) → *γ*_*B*1_ *I*_BB_**/*μ*_*B*_ = *R*_BB_** as *t* → *∞*. The proof is concluded using similar arguments as in the proof of Theorem 6.

The epidemiological implication of Theorem 8 is that BTB will be endemic in the buffalo population if ${\tilde{R}}_{0}>1$ (and *θ*_*EB*_ = *θ*_*RB*_ = *δ*_*B*_ = 0).  Figure 4(b) depicts the solutions of model (27) for the case when ${\tilde{R}}_{0}>1$ and *θ*_*EB*_ = *θ*_*RB*_ = *δ*_*B*_ = 0, showing convergence of the initial solutions to the unique endemic equilibrium (in line with Theorem 8).

### 3.4. Sensitivity and Uncertainty Analyses

In this section, sensitivity and uncertainty analyses will be carried out, using Latin hypercube sampling (LHS) and partial correlation coefficient (PRCC) [39–41], to assess the effect of uncertainty in the estimate of the parameter values used to simulate the buffalo-only model (on the simulation results obtained) and to determine the key parameters that drive the dynamics of the disease in the buffalo-human population. The ranges and baseline values of the parameters of the buffalo-only model, given in Table 3 with *θ*_*RB*_ = *θ*_*EB*_ = 0 (i.e., in the absence of backward bifurcation), will be used in these analyses. Each parameter of the buffalo-only model (22) is assumed to obey a uniform distribution [42]. Following [42], a total of 1000 LHS runs (*N* = 1000) are carried out. Furthermore, the following initial conditions (which are consistent with the dynamics of African buffalo in the Kruger National Park [29]): (*S*_*B*_(0), *E*_*B*1_(0), *E*_*M*1_(0), *E*_*B*2_(0), *E*_*M*2_(0), *I*_*BB*_(0), *I*_*MB*_(0), *R*_*BB*_(0), *R*_*M*B_(0)) = (28000, 100, 100, 20, 20, 10, 10, 100, 100)) are used in the simulations.

**Figure 5:**
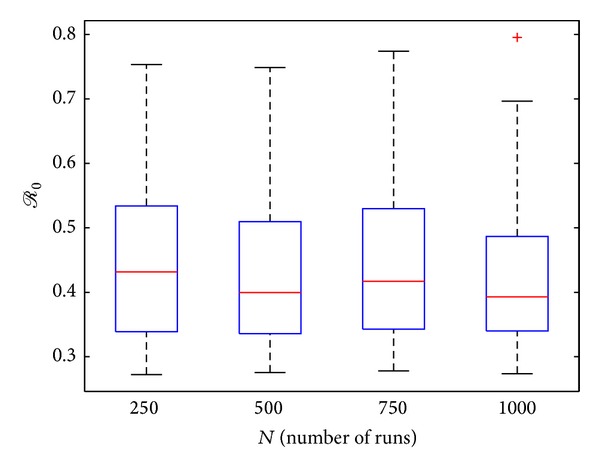
Box plot of *R*_0_ as a function of the number of LHS runs carried out for the buffalo-only model (22), using parameter values and ranges given in Table 3 with *θ*_*EB*_ = *θ*_*RB*_ = 0.

**Figure 6:**
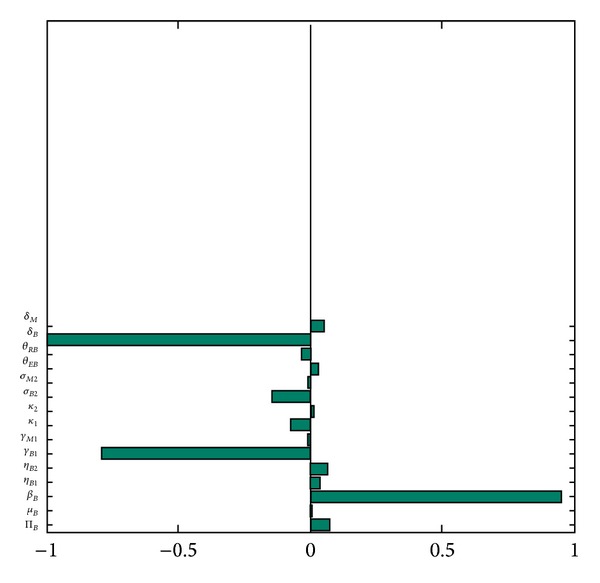
PRCC values of the parameters of the buffalo-only model (22), using *R*_0_ as the output function. Parameter values used are as given in Table 3.

Figure 5 depicts a box plot of *R*_0_, as a function of the number of LHS runs carried out (*N* = 1000), from which it is evident that the distribution of *R*_0_ lies in the range *R*_0_ ∈ [0.25, 0.75] (each box plot displays the upper and lower quartile ranges of *R*_0_. a horizontal line within the box is the median value, and values of *R*_0_ beyond the whiskers are outliers [43]). Thus, since the distribution of the reproduction number of the buffalo-only model is less than unity, it follows (from Theorem 6) that the BTB-MTB outbreaks (in the buffalo-human population) will die out with time (in other words, the disease will be effectively controlled). The PRCC values of the parameters of the buffalo-only model (22), using *R*_0_ as the response function, are depicted in Figure 6. It follows from Figure 6 that the top three parameters that most influences the value of *R*_0_ (hence the disease dynamics) are the BTB transmission rate (*β*_*B*_), the recovery rate of buffalos (*γ*_*B*1_), and the BTB-induced mortality in buffalos (*δ*_*B*_).

**Figure 7:**
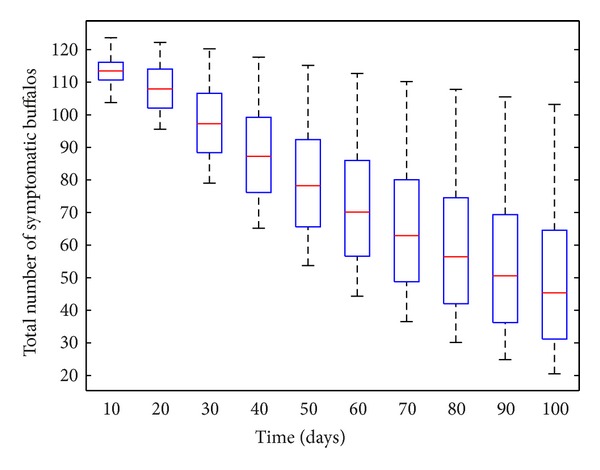
Box plot of the total number of symptomatic buffalos (*I*_*BB*_ + *I*_*MB*_) as a function of the number of LHS runs for the buffalo-only model (22), using parameter values and ranges given in Table 3 with *θ*_*EB*_ = *θ*_*RB*_ = 0.

**Figure 8:**
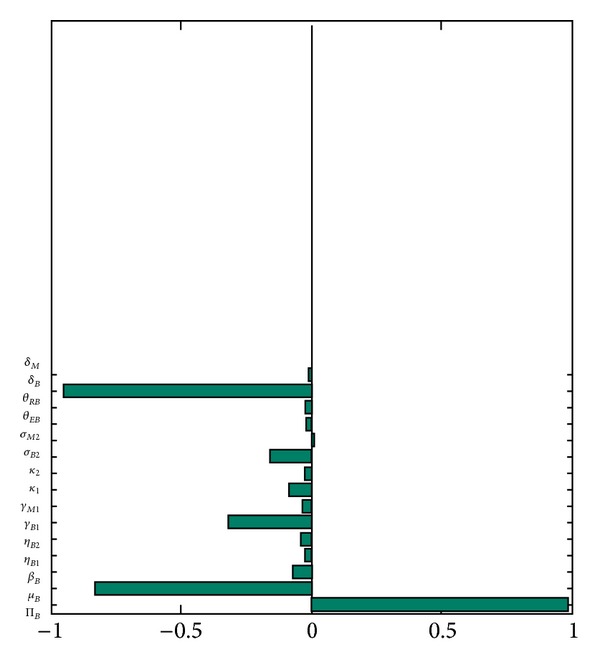
PRCC values of the parameters of the buffalo-only model (22), using total number of symptomatic buffalos (*I*_*BB*_ + *I*_*MB*_) as the output function. Parameter values used are as given in Table 3.

Similarly, Figure 7 depicts the box plot of the buffalo-only model (22) using total number of symptomatic buffalos (*I*_*BB*_ + *I*_*MB*_) as the response function. This figure shows a distribution of the number of symptomatic buffalos lying in the range [20–130]. Hence, this study shows that, using the parameter values and ranges relevant to BTB-MTB dynamics at the Kruger National Park, a BTB outbreak could cause no more than 130 confirmed cases (of BTB and MTB) in the park. The associated PRCC values (with the total number of symptomatic buffalos as the output) are depicted in Figure 8, from which it is evident that, in this scenario, the top three parameters (that most influences the output) are the buffalo recruitment rate (Π_*B*_), the natural (*μ*_*B*_), and the disease-induced (*δ*_*B*_) death rate of buffalos. Hence, this study shows variability in the top-ranked PRCC values on the chosen response/output function.

Having fully studied the dynamics of the bovine-only model (22), the full BTB-MTB model (22) will now be analysed.

## 4. Analysis of the BTB-MTB Model

It can be shown, using the approach in Section 3, that the following biologically feasible region

(61) Ω={(SH,EH1,EH2,IH1,IH2,RH1,RH2,SB,EB1,EM1,EB2,EM2,IBB,IMB,RBB,RMB)∈R+16:NH≤ΠHμH,NB≤ΠBμB}

is positively invariant and attracting for the BTB-MTB model (21).

### 4.1. Local Stability of DFE

The analyses in this section will be carried out for the special case of the BTB-MTB model (21) with *θ*_*MM*_ = *θ*_*BB*_ = 0. The justification for this assumption is based on the fact that contact between humans and buffalos in the Kruger National Park are tightly controlled (hence, it is reasonable to assume that buffalo-to-human or human-to-buffalo transmission of BTB is negligible). The DFE of the BTB-MTB model (21) is given by

(62) E0f=(SH∗,EH1∗,EH2∗,IH1∗,IH2∗,RH1∗,RH2∗,SB∗,EB1∗,EM1∗,EB2∗,EM2∗,IBB∗,IMB∗,RBB∗,RMB∗)=(ΠHμH,0,0,0,0,0,0,ΠBμB,0,0,0,0,0,0,0,0).

The associated next generation matrices of the BTB-MTB model (21), denoted by *F*_*f*_ and *V*_*f*_, are given, respectively, by

(63) Ff=[βHηH10βH00000000βHηH20βH000000000000000000000000000000βBηB10βBηB20βB000000000000000000000000000000000000000000000000000],Vf=[Q10000000000Q200000000−σ10Q300000000−σ20Q40000000000K10000000000K200000000−κ10K3000000000−κ2K400000000−σB20K500000000−σM20K6].

It follows then that the* reproduction number* of the BTB-MTB model (21), denoted by *R*_*f*_, is given by

(64) $$\begin{array}{c} R_{f}=\rho \left(F_{f}{V_{f}}^{-1}\right)=\max\left\{R_{HM},R_{HB},R_{0}\right\}, \end{array}$$

where *R*_*HM*_ and *R*_*HB*_ are the associated* reproduction numbers* for humans infected with MTB and with BTB, respectively, given by

(65) $$\begin{array}{c} R_{HM}=\frac{\beta_{H}\left(\eta_{H1}Q_{3}+\sigma_{1}\right)}{Q_{1}Q_{3}},\;\;R_{HB}=\frac{\beta_{H}\left(\eta_{H2}Q_{4}+\sigma_{2}\right)}{Q_{2}Q_{4}}, \end{array}$$

where *Q*_1_ = *σ*_1_ + *μ*_*H*_, *Q*_2_ = *σ*_2_ + *μ*_*H*_, *Q*_3_ = *γ*_1_ + *μ*_*H*_ + *δ*_*H*1_, and *Q*_4_ = *γ*_2_ + *μ*_*H*_ + *δ*_*H*2_, and *R*_0_ is as defined in Section 3. Thus, using the approach in Section 3.1, the following result can be established for the BTB-MTB model (21).

**Lemma 9 .**

The DFE, *E*_0*f*_, of model (21), with *θ*_*MM*_ = *θ*_*BB*_ = 0, is LAS in *Ω* if *R*_*f*_ < 1, and unstable if *R*_*f*_ > 1.

It can be shown, as in Section 3, that the BTB-MTB model (21) also undergoes backward bifurcation. Unlike in the buffalo-only model (22), however, this phenomenon persists even if the bovine-associated reinfection terms (*θ*_*RB*_ and *θ*_*EB*_) are set to zero. This is due to the reinfection of exposed and recovered humans (i.e., *θ*_*H*1_ ≠ 0 and *θ*_*H*2_ ≠ 0). To illustrate this fact, it is shown that the DFE (*E*_0*f*_) of the BTB-MTB model (21) is GAS in *Ω* in the absence of reinfection of exposed and recovered buffalos and humans, whenever the associated reproduction number (*R*_*f*_) is less than unity.

### 4.2. Global Asymptotic Stability of DFE

**Theorem 10 .**

The DFE, *E*_0*f*_, of the BTB-MTB model (21) with *θ*_*H*1_ = *θ*_*H*2_ = *θ*_*RB*_ = *θ*_*RH*_ = *θ*_*BB*_ = *θ*_*MM*_ = *θ*_*EB*_ = *θ*_*RB*_ = 0, is GAS in *Ω* if *R*_*f*_ < 1.

**Proof**

The proof of Theorem 10, based on using comparison theorem [44], is given in Appendix B. Hence, the analyses in this section show that the buffalo-only model and the full BTB-MTB model (21) have essentially the same qualitative dynamics with respect to the local and global asymptotic stability of the associated disease-free equilibrium (in the absence of reinfection) as well as the backward bifurcation property established in the transmission dynamics of BTB and BTB-MTB in a buffalo-human population. In both cases, the backward bifurcation phenomenon is shown to arise due to the reinfection of the exposed and recovered host(s) (buffalos for the buffalo-only model (22), and buffalos and humans for the BTB-MTB model).

### 4.3. Numerical Simulations

The BTB-MTB model (21) is simulated, using the baseline values tabulated in Table 3 (unless otherwise stated), to assess the effect of the dynamics of BTB (MTB) on the spread of MTB (BTB) in the human (buffalo) population.

#### 4.3.1. Effect of BTB on MTB

The effect of BTB (in the human-buffalo population within the Kruger National Park) on the spread of MTB in the human population (within the park) is assessed by simulating the BTB-MTB model (21) using parameter values in Table 3, subject to the following four effectiveness levels of BTB transmission likelihood from buffalos to humans (i.e., choosing four different values of the parameter *θ*_*MM*_, for the reduced likelihood of humans acquiring BTB infection from buffalos):

- no transmission of BTB from buffalos to humans: *θ*_*MM*_ = 0,
- low rate of transmission of BTB from buffalos to humans: *θ*_*MM*_ = 0.25,
- moderate rate of transmission of BTB from buffalos to humans: *θ*_*MM*_ = 0.50,
- high rate of transmission of BTB from buffalos to humans: *θ*_*MM*_ = 0.75.

**Figure 9:**
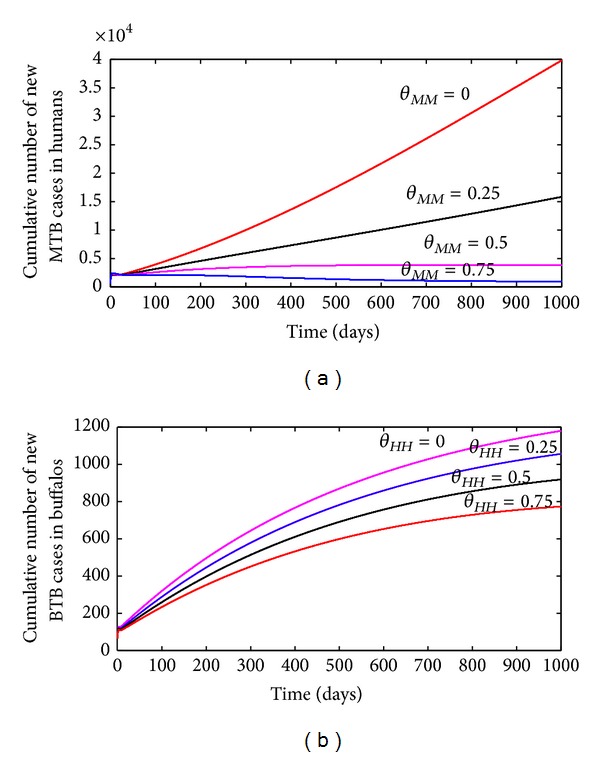
Cumulative number of new cases of (a) MTB infection in humans and (b) BTB infection in buffalos. Parameter values used are as given in Table 3, with various values of *θ*_*MM*_ (a) or *θ*_*HH*_ (b).

The simulation results obtained, depicted in Figure 9(a), show that the cumulative number of new MTB cases in humans decreases with increasing rate of BTB transmission to humans by buffalos (*θ*_*MM*_).

#### 4.3.2. Effect of MTB on BTB

Similar plot is generated to assess the effect of MTB (in the human-buffalo population) on the spread of BTB in the buffalo population. Here, too, four transmission levels of the associated parameter (*θ*_*HH*_) are considered, namely, none (*θ*_*HH*_ = 0), low (*θ*_*HH*_ = 0.25), moderate (*θ*_*HH*_ = 0.50), and high (*θ*_*HH*_ = 0.75). The results obtained, depicted in Figure 9(b), show that the cumulative number of new BTB infections in buffalos decreases with increasing rate of MTB transmission to buffalos by humans.

## 5. Conclusions

A new deterministic model for the transmission dynamics of BTB and MTB in a community of humans and buffalos is designed and rigorously analyzed. Some of the main findings of the study are as follows.

- The buffalo-only model undergoes the phenomenon of backward bifurcation. This phenomenon is caused by the exogenous reinfection of exposed and infected buffalos. In the absence of reinfection, the disease-free equilibrium of the buffalo-only model is shown to be globally asymptotically stable whenever the associated reproduction number of the model is less than unity.
- In the absence of the reinfection of exposed and recovered buffalos (*θ*_*EB*_ = *θ*_*RB*_ = 0), the buffalo-only model is shown to have unique endemic equilibrium whenever its reproduction number exceeds unity (*R*_0_ > 1). This equilibrium is shown to be globally asymptotically stable for the special case where the disease-induced mortality in buffalos is negligible (*δ*_*B*_ = 0).
- Detailed uncertainty analyses of the buffalo-only model, using a reasonable set of parameter values and ranges (Table 3) relevant to BTB dynamics in the Kruger National Park, shows that the distribution of the associated reproduction number of the buffalo-only model is less than unity (hence, BTB outbreaks will not persist in the Park). Furthermore, such outbreak would cause no more than 120 confirmed (symptomatic) cases of BTB or MTB within the Park. Sensitivity analysis, for the case when the reproduction number (*R*_0_) is chosen as the response/output function, reveals that the three main parameters that govern the disease dynamics are the BTB transmission rate, recovery rate of buffalos, and BTB-induced mortality rate. Similarly, three parameters (recruitment rate of buffalos, natural, and BTB-induced death rates in buffalos) are identified as the main influential parameters for the case where the number of symptomatic buffalos (with BTB or MTB) is the chosen output function.
- The BTB-MTB model also undergoes backward bifurcation. Unlike in the buffalo-only model, this phenomenon persists even if the bovine-associated reinfection terms (*θ*_*RB*_ and *θ*_*EB*_) are set to zero. This is due to the reinfection of exposed and recovered humans (*θ*_*H*1_ ≠ 0, *θ*_*H*2_ ≠ 0, *θ*_*RH*_ ≠ 0, *θ*_*RB*_ ≠ 0). It is shown that this model does not undergo backward bifurcation in the absence of reinfection of exposed and recovered host(s) (buffalos and humans). For this case, it is shown that the DFE of the BTB-MTB model (21) is globally asymptotically stable, whenever the associated reproduction number is less than unity.
- The buffalo-only model and the full BTB-MTB model exhibit the same qualitative dynamics with respect to the local and global asymptotic stability of the associated disease-free equilibrium (in the absence of reinfection of the associated host(s)). In both models, the backward bifurcation phenomenon is shown to arises due to the reinfection of exposed and recovered host(s).
- Numerical simulations of the BTB-MTB model show that an increase in the cumulative number of BTB infection leads to a marked reduction in the cumulative number of new MTB cases in humans. Similarly, an increase in the cumulative number of MTB infection led to a significant decrease in the cumulative number of new BTB cases in buffalos.

## Figures and Tables

**Table 1:** Description of the variables of the BTB-MTB model (21).

Variable	Interpretation
*S*_*H*_	Population of susceptible humans
*E*_*H*1_	Population of humans exposed to MTB
*E*_*H*2_	Population of humans exposed to BTB
*I*_*H*1_	Population of infected humans with clinical symptoms of MTB
*I*_*H*2_	Population of infected humans with clinical symptoms of BTB
*R*_*H*1_	Population of humans who recovered from MTB
*R*_*H*2_	Population of humans who recovered from BTB
*S*_*B*_	Population of susceptible buffalos
*E*_*B*1_	Population of buffalos early-exposed to BTB
*E*_*M*1_	Population of buffalos early-exposed to MTB
*E*_*B*2_	Population of buffalos at advanced-exposed BTB stage
*E*_*M*2_	Population of buffalos at advanced-exposed MTB stage
*I*_*BB*_	Population of buffalos with clinical symptoms of BTB
*I*_*MB*_	Population of buffalos with clinical symptoms of MTB
*R*_*BB*_	Population of buffalos who recovered from BTB
*R*_*MB*_	Population of buffalos who recovered from MTB

**Table 2:** Description of parameters of the BTB-MTB model (21).

Parameter	Interpretation
Π_*H*_	Recruitment rate of humans
Π_*B*_	Recruitment rate of buffalos
*μ*_*H*_	Natural death rate of humans
*μ*_*B*_	Natural death rate of buffalos
*β*_*H*_	Transmission rate of MTB
*β*_*B*_	Transmission rate of BTB
*η*_*H*1_	Modification parameter for the reduction in infectiousness of exposed humans in comparison to humans with clinical symptoms of MTB
*η*_*H*2_	Modification parameter for the reduction in infectiousness of exposed humans in comparison to humans with clinical symptoms of BTB
*η*_*B*1_, *η*_*B*2_	Modification parameters for the reduction in infectiousness of exposed buffalos in comparison to buffalos with clinical symptoms of BTB
*θ*_*HH*_, *θ*_*BB*_	Modification parameters for the reduction in transmissibility of MTB to buffalos in comparison to humans
*θ*_*MM*_	Modification parameters for the reduction in transmissibility of BTB to humans in comparison to buffalos
*γ*_*i*_ (*i* = 1, 2)	Recovery rate of humans
*γ*_*B*1_, *γ*_*M*1_	Recovery rate of buffalos
*σ*_*i*_ (*i* = 1, 2)	Progression rate from *E*_*Hi*_ to *I*_*Hi*_ class
*κ*_1_	Progression rate from *E*_*B*1_ to *E*_*B*2_ class
*κ*_2_	Progression rate from *E*_*M*1_ to *E*_*M*2_ class
*σ*_*B*2_	Progression rate from *E*_*B*2_ to *I*_*BB*_ class
*σ*_*M*2_	Progression rate from *E*_*M*2_ to *I*_*MB*_ class
*θ*_*Hi*_ (*i* = 1, 2)	Exogenous reinfection rate for humans in the *E*_*Hi*_ class
*θ*_*RB*_, *θ*_*RH*_	Exogenous reinfection rate for recovered humans
*θ*_*EB*_	Exogenous reinfection rate for buffalos in the exposed and recovered classes, respectively
*δ*_*H*1_, *δ*_*H*2_	Disease-induced death rate for humans
*δ*_*B*_, *δ*_*M*_	Disease-induced death rate for buffalos

**Table 3:** Ranges and baseline values for parameters of the BTB-MTB model (21).

Parameter	Range (day^−1^)	Baseline value (day^−1^)	Reference
Π_*H*_	[26, 80]	53	[20]
Π_*B*_	[2, 4]	3	[20–22]
*μ*_*H*_	[0.0000274, 0.0000549]	0.000047	[14, 23–25]
*μ*_*B*_	(0.00009477, 0.00011583)	0.0001053	[22, 26]
*β*_*H*_	[0.00011, 0.000959]	0.000535	[14, 17]
*β*_*B*_	(0.006597, 0.008063)	0.00733	[10]
*η*_*H*1_	[0, 1)	0.5	Fitted
*η*_*H*2_	[0, 1)	0.5	Fitted
*η*_*B*1_	(0.4455, 0.5045)	0.45	Fitted
*η*_*B*2_	(0.495, 0.605)	0.55	Fitted
*θ*_*BB*_	[0, 1)	0.5	Fitted
*θ*_*MM*_	[0, 1]	0.5	Assumed
*θ*_*HH*_	[0, 1)	0.5	Assumed
*γ*_*i*_ (*i* = 1, 2)	(0.0000823, 0.000823)	0.000453	[14, 23]
*γ*_*B*1_	(0.00774, 0.00946)	0.0086	[10]
*γ*_*M*1_	(0.13374, 0.160086)	0.1486	[12]
*σ*_*i*_ (*i* = 1, 2)	(0.0000822, 0.00247)	0.001	[23, 27]
*κ*_1_	(0.45, 0.55)	0.5	[27]
*κ*_2_	(0.45, 0.55)	0.5	[27]
*σ*_*B*2_	(0.25, 0.35)	0.3	[27]
*σ*_*M*2_	(0.36, 0.44)	0.4	[27]
*θ*_*Hi*_ (*i* = 1, 2)	[0, 0.1]	0.00271	[23]
*θ*_*RB*_, *θ*_*RH*_	[0.002439, 0.002981]	0.00271	[23]
*θ*_*EB*_	[0.002439, 0.002981]	0.00271	[23]
*δ*_*H*1_, *δ*_*H*2_	[0.000115, 0.000822]	0.0002	[14, 17, 23, 25]
*δ*_*B*_	[0.0018, 0.0022]	0.002	[28]
*δ*_*M*_	[0.0018, 0.0022]	0.002	[14, 23]

**Table 4:** Number of symptomatic buffalos with BTB at Kruger National Park [29].

Year	Number of symptomatic buffalos [29]
2001	35
2002	135
2003	185
2004	238
2005	230

## Appendices

### A. Proof of Theorem 4

Proof The proof is based on using centre manifold theory [17, 33]. Consider the buffalo-only model (22). Let *S*_*I*_ = *x*_1_, *E*_*B*1_ = *x*_2_, *E*_*M*1_ = *x*_3_, *E*_*B*2_ = *x*_4_, *E*_*M*2_ = *x*_5_, *I*_*BB*_ = *x*_6_, *I*_*MB*_ = *x*_7_, *R*_*BB*_ = *x*_8_, and *R*_*MB*_ = *x*_9_. Thus, *N*_*B*_ = ∑_*i*=1_^9^ *x*_*i*_. Further, by using the vector notation **X** = (*x*_1_, *x*_2_, *x*_3_, *x*_4_, *x*_5_, *x*_6_, *x*_7_, *x*_8_, *x*_9_)^*T*^, the buffalo-only model (22) can be written in the form *d* **X**/*dt* = (*f*_1_, *f*_2_, *f*_3_, *f*_4_, *f*_5_, *f*_6_, *f*_7_, *f*_8_, *f*_9_)^*T*^ as follows:

(A.1) dx1dt=ΠB−(λB+μB)x1,dx2dt=λBx1−(θEBλB+κ1+μB)x2,dx3dt=−(κ2+μB)x3,dx4dt=κ1x2−(θEBλB+σB2+μB)x4,dx5dt=κ2x3−(σM2+μB)x5,dx6dt=σB2x4+(x2+x4)θEBλB+θRBλBx8−(γB1+μB+δB)x6,dx7dt=σM2x5−(γM1+μB+δB)x7,dx8dt=γB1x6−(θRBλB+μB)x8,dx9dt=γM1x7−μBx9,

with the associated force of infection given by

(A.2) $$\begin{array}{c} \lambda_{B}=\frac{\beta_{B}\left(\eta_{B1}x_{2}+\eta_{B2}x_{4}+x_{6}\right)}{\sum_{i=1}^{9}x_{i}}. \end{array}$$

Consider the case with *R*_0_ = 1. Let *β*_*B*_* (obtained by solving for *β*_*B*_ = *β*_*B*_* from *R*_0_ = 1), given by

(A.3) $$\begin{array}{c} \beta_{B}=\beta_{B}^{*}=\frac{K_{1}K_{3}K_{5}}{\eta_{B1}K_{3}K_{5}+\kappa_{1}\left(\eta_{B2}K_{5}+\sigma_{B2}\right)}, \end{array}$$

be chosen as a bifurcation parameter. The Jacobian of the system (A.1), evaluated at the DFE (*E*_0_) with *β*_*B*_ = *β*_*B*_* (denoted by *J**), is given by

(A.4) $$\begin{array}{c} J^{*}=\left[\begin{array}{ccccccccc} -\mu_{B} & -\beta_{B}^{*}\eta_{B1} & 0 & -\beta_{B}^{*}\eta_{B2} & 0 & -\beta_{B}^{*} & 0 & 0 & 0\\ 0 & \beta_{B}^{*}\eta_{B1}-K_{1} & 0 & \beta_{B}^{*}\eta_{B2} & 0 & \beta_{B}^{*} & 0 & 0 & 0\\ 0 & 0 & -K_{2} & 0 & 0 & 0 & 0 & 0 & 0\\ 0 & \kappa_{1} & 0 & -K_{3} & 0 & 0 & 0 & 0 & 0\\ 0 & 0 & \kappa_{2} & 0 & -K_{4} & 0 & 0 & 0 & 0\\ 0 & 0 & 0 & \sigma_{B2} & 0 & -K_{5} & 0 & 0 & 0\\ 0 & 0 & 0 & 0 & \sigma_{M2} & 0 & -K_{6} & 0 & 0\\ 0 & 0 & 0 & 0 & 0 & \gamma_{B1} & 0 & -\mu_{B} & 0\\ 0 & 0 & 0 & 0 & 0 & 0 & \gamma_{M1} & 0 & -\mu_{B} \end{array}\right], \end{array}$$

where *K*_*i*_  (*i* = 1,…, 6) are as defined in Section 3. The Jacobian (*J**) of the linearized system has a simple zero eigenvalue (with all other eigenvalues having negative real part). Hence, the centre manifold theory [17, 33] can be used to analyse the dynamics of the system (A.1) around *β*_*B*_ = *β*_*B*_*. Using the notation in [17], the following computations are carried out.
Eigenvectors of *J**|_*β*_*B*_=*β*_*B*_*_
For the case when *R*_0_ = 1, it can be shown that the Jacobian, *J**, has a right eigenvector (corresponding to the simple zero eigenvalue), given by **w** = [*w*_1_, *w*_2_, *w*_3_, *w*_4_, *w*_5_, *w*_6_, *w*_7_, *w*_8_, *w*_9_]^*T*^, where

(A.5) $$\begin{array}{c} w_{1}=\frac{-\beta_{B}^{*}\left(\eta_{B1}w_{2}+\eta_{B2}w_{4}+w_{6}\right)}{\mu },\\ w_{2}=w_{2},\;\;w_{3}=0,\\ w_{4}=\frac{K_{5}w_{6}}{\sigma_{B2}},\;\;w_{5}=\frac{K_{6}w_{7}}{\sigma_{M2}},\\ w_{6}=w_{6},\;\;w_{7}=w_{7},\\ w_{8}=\frac{\gamma_{B1}w_{6}}{\mu_{B}},\;\;w_{9}=\frac{\gamma_{M1}w_{7}}{\mu_{B}}. \end{array}$$

Similarly, the components of the left eigenvector of *J** (corresponding to the simple zero eigenvalue), denoted by **v** = [*v*_1_, *v*_2_, *v*_3_, *v*_4_, *v*_5_, *v*_6_, *v*_7_, *v*_8_, *v*_9_], are given by

(A.6) v3=κ2σM2v7K2K4,v4=(K1−βB∗ηB1)v2κ1=1K3K5[βB∗v2(ηB2K5+σB2)+σB2γB1v8],v5=σM2v7K4,  v6=βB∗v2+γB1v8K5,  v9=K6v7γM1,v1=0,   v2>0,   v7>0,   v8>0.

It is worth mentioning that the free right eigenvectors, *w*_2_, *w*_6_, and *w*_7_ and left eigenvectors, *v*_2_, *v*_7_, and *v*_8_, are chosen to be

(A.7) v2=1,  v7=1K6,  v8=1,w2=13,  w6=13A1,  w7=13A2,

where

(A.8) A1=[βB∗(ηB2K5+σB2)+γB1σB2]K3σB2 +μB(βB∗+γB1)+γB1K5K5μB,A2=K2[μB(K4+K6)+K4K6]K2K4K6μB,

so that **v** · **w** = 1 (in line with [17]). It can be shown, by computing the nonzero partial derivatives of the right-hand side functions, *f*_*i*_  (*i* = 1,…, 9), that the associated backward bifurcation coefficients, *a* and *b*, are given, respectively, by (see Theorem  4.1 in [17])

(A.9) a=∑k,i,j=18vkwiwj∂2fk∂xi∂xj(0,0),=2βB∗μBΠB{θEB[w2(v6−v2)+w4(v6−v4)] +θRBw8(v6−v8)−v2(w2+w3+w4+w5+w6+w7+w8+w9)},=2βB∗μB3ΠB{θEB(βB∗+γB1−K5K5+A3)+θRBA1(βB∗+γB1−K5K5)−[1+μBK5+σB1+σB2γB1A1μBσB2+γM1σM2K2+K2μBK6+σB2A2K2μBσM2]},b=∑k,i=19vkwi∂2fk∂xi∂βB∗(0,0)=v2(ηB1w2+ηB2w4+w6)=13[ηB1+1A1σB2(ηB2K5+σB2)],

where

(A.10) A3=1A1σB2K3[K3(βB∗+γB1)−βB∗(ηB2K5+σB2)+σB2γB1].

Since the bifurcation coefficient, *b*, is automatically positive, it follows from Theorem  4.1 in [17] that the buffalo-only model (22) (or its transformed equivalent (A.1)) will undergo backward bifurcation if the bifurcation coefficient, *a*, given by (A.9), is positive.

### B. Proof of Theorem 10

Proof Consider BTB-MTB model (22) with with *θ*_*H*1_ = *θ*_*H*2_ = *θ*_*RB*_ = *θ*_*RH*_ = *θ*_*BB*_ = *θ*_*MM*_ = *θ*_*EB*_ = *θ*_*RB*_ = 0. The proof is based on using a comparison theorem [44]. It should be noted, first of all, that the equations for the infected components in the BTB-MTB model (21) can be rewritten in the following matrix form:

(B.1) $$\begin{array}{c} \frac{d\tilde{x}}{dt}=\left[\left(F-V\right)-\left(1-\frac{S_{H}}{N_{H}}\right)M_{1}-\left(1-\frac{S_{B}}{N_{B}}\right)M_{2}\right]\tilde{x}, \end{array}$$

where $\tilde{x}={\left[E_{H1},E_{H2},I_{H1},I_{H2},E_{B1},E_{M1},E_{B2},E_{M2},I_{BB},I_{MB}\right]}^{T}$, the matrices *F*_*f*_ and *V*_*f*_ are as given in Section 4, and

(B.2) M1=[βHηH10βH00000000βHηH20βH00000000000000000000000000000000000000000000000000000000000000000000000000000000000000],M2=[00000000000000000000000000000000000000000000βBηB10βBηB20βB000000000000000000000000000000000000000000000000000].

It follows, since *S*_*H*_(*t*) < *N*_*H*_(*t*) and *S*_*B*_(*t*) < *N*_*B*_(*t*) for all *t* ≥ 0 in *Ω*, that

(B.3) $$\begin{array}{c} \frac{d\tilde{x}}{dt}\leq \left(F-V\right)\tilde{x}. \end{array}$$

Using the fact that the eigenvalues of the matrix *F*_*f*_ − *V*_*f*_ all have negative real parts (where *ρ*(*F*_*f*_ *V*_*f*_^−1^) < 1 if *R*_*f*_ < 1, which is equivalent to *F*_*f*_ − *V*_*f*_ having eigenvalues with negative real parts when *R*_*f*_ < 1 of [33]), consequently, the linearized differential inequality system (B.3) is stable whenever *R*_*f*_ < 1. Thus,

(B.4) (EH1(t),EH2(t),IH1(t),IH2(t), EB1(t),EM1(t),EB2(t),EM2(t),IBB(t),IMB(t)) ⟶(0,0,0,0,0,0,0,0,0,0) as  t→∞.

It follows, by comparison theorem (see [44], pp 31), that

(B.5) (EH1(t),EH2(t),IH1(t),IH2(t),EB1(t),EM1(t),EB2(t),EM2(t),IBB(t),IMB(t)) ⟶(0,0,0,0,0,0,0,0,0,0).

Substituting  *E*_*H*1_(*t*)  =  *E*_*H*2_(*t*)  =  *I*_*H*1_(*t*)  =   *I*_*H*2_(*t*)   =  *E*_*B*1_(*t*) =  *E*_*M*1_(*t*)  =  *E*_*B*2_(*t*)  =  *E*_*M*2_(*t*)  =  *I*_*BB*_(*t*) = *I*_*MB*_(*t*) = 0 in the susceptible and the recovered compartments of (21) gives *S*_*H*_(*t*) → *S*_*H*_*, *R*_*H*1_ → 0, *R*_*H*2_ → 0, *S*_*B*_(*t*) → *S*_*B*_*, *R*_*BB*_ → 0, and *R*_*MB*_ → 0 as *t* → *∞*. Thus, the DFE (*E*_0*f*_) of the BTB-MTB model (21) is GAS in *Ω* if *R*_*f*_ < 1 and with *θ*_*H*1_ = *θ*_*H*2_ = *θ*_*RB*_  =  *θ*_*RH*_ = *θ*_*BB*_  =  *θ*_*MM*_ = *θ*_*EB*_ = *θ*_*RB*_ = 0.

## References

[1] Centers for Disease Control http://www.cdc.gov/tb/publications/factsheets/general/mbovis.pdf.

[2] Grange JM, Collins CH (1987). Bovine tubercle bacilli and disease in animals and man. *Epidemiology and Infection*.

[3] de Lisle GW, Mackintosh CG, Bengis RG (2001). Mycobacterium bovis in free-living and captive wildlife, including farmed deer. *Revue Scientifique et Technique de L'Office International des Epizooties*.

[4] WHO http://www.who.int/tb/publications/global_report/en/.

[5] Frye G, Thoen CO, Steele JH (1994). Bovine tuberculosis eradication. *Mycobacterium Bovis Infection in Animals and Humans*.

[6] http://www.cfsph.iastate.edu/Factsheets/pdfs/bovine_tuberculosis.pdf.

[7] Ayele WY, Neill SD, Zinsstag J, Weiss MG, Pavlik I (2004). Bovine tuberculosis: an old disease but new threat to Africa. *International Journal of Tuberculosis and Lung Disease*.

[8] de Vos V, Bengis RG, Kriek NPJ (2001). The epidemiology of tuberculosis in free-ranging African buffalo (*Syncerus caffer*) in the Kruger national park, South Africa. *Journal of Veterinary Research*.

[9] Etter E, Donado P, Jori F, Caron A, Goutard F, Roger F (2006). Risk analysis and bovine tuberculosis, a re-emerging zoonosis. *Annals of the New York Academy of Sciences*.

[10] Cross PC, Getz WM (2006). Assesing vaccination as a control strategy in an on going epidemic: bovine tuberculosis in African buffalo. *Ecological Modelling*.

[11] Herd of Cape Buffalo, Kruger National Park. http://www.krugerpark.co.za/africa_african_buffalo.html.

[12] Adewale SO, Podder CN, Gumel AB (2009). Mathematical analysis of a TB transmission model with DOTS. *Canadian Applied Mathematics Quarterly*.

[13] Agusto FB, Lenhart S, Gumel AB, Odoi A (2011). Mathematical analysis of a model for the transmission dynamics of bovine tuberculosis. *Mathematical Methods in the Applied Sciences*.

[14] Bhunu CP, Garira W, Mukandavire Z, Zimba M (2008). Tuberculosis transmission model with chemoprophylaxis and treatment. *Bulletin of Mathematical Biology*.

[15] Bishai WR, Graham NM, Harrington S (1998). Molecular and geographic patterns of tuberculosis transmission after 15 years of directly observed therapy. *The Journal of the American Medical Association*.

[16] Anguelov R, Kojouharov H Continuous age-structured model for bovine tuberculosis in African buffalo.

[17] Castillo-Chavez C, Song B (2004). Dynamical models of tuberculosis and their applications. *Mathematical Biosciences and Engineering*.

[18] Kao RR, Roberts MG, Ryan TJ (1997). A model of bovine tuberculosis control in domesticated cattle herds. *Proceedings of the Royal Society B: Biological Sciences*.

[19] van den Driessche P, Wang L, Zou X (2007). Modeling diseases with latency and relapse. *Mathematical Biosciences and Engineering*.

[20] South African National Parks (SANParks)

[21] Michel AL, Bengis RG, Keet DF (2006). Wildlife tuberculosis in South African conservation areas: implications and challenges. *Veterinary Microbiology*.

[22] Wessel CO (2006). *Chemical Immobilization of African Buffalo (Syncerus caffer) in Kruger National Park: Evaluating effects on survival and reproduction*.

[23] Cohen T, Colijn C, Finklea B, Murray M (2007). Exogeneous re-infection and the dynamics of tuberculosis epidemics: local effects in a network model transmission. *Journal of the Royal Society Interface*.

[24] Dye C, Williams BG (2008). Eliminating human tuberculosis in the twenty-first century. *Journal of the Royal Society Interface*.

[25] Feng Z, Castillo-Chavez C, Capurro AF (2000). A model for tuberculosis with exogenous reinfection. *Theoretical Population Biology*.

[26] Cronje HP, Reilly BK, Macfadyen ID (2002). Natural mortality among four common ungulate species on Letaba Ranch, Limpopo Province, South Africa. *Koedoe*.

[27] Resch SC, Salomon JA, Murray M, Weinstein MC (2006). Cost-effectiveness of treating multidrug-resistant tuberculosis. *PLoS Medicine*.

[28] Cross PC, Lloyd-Smith JO, Bowers JA, Hay CT, Hofmeyr M, Getz WM (2004). Integrating association data and disease dynamics in a social ungulate: bovine tuberculosis in African buffalo in the Kruger National Park. *Annales Zoologici Fennici*.

[29] Cross PC, Heisey DM, Bowers JA (2009). Disease, predation and demography: assessing the impacts of bovine tuberculosis on African buffalo by monitoring at individual and population levels. *Journal of Applied Ecology*.

[30] Lay G, Poquet Y, Salek-Peyron P (2007). Langhans giant cells from *M. Tuberculosis*-induced human granulomas cannot mediate mycobacterial uptake. *Journal of Pathology*.

[31] Hethcote HW (2000). The mathematics of infectious diseases. *SIAM Review*.

[32] Diekmann O, Heesterbeek JAP, Metz JAJ (1990). On the definition and computation of the basic reproductive ratio *R*
_0_ in model for infectious diseases in heterogeneous population. *Journal of Mathematical Biology*.

[33] van den Driessche P, Watmough J (2002). Reproduction numbers and sub-threshold endemic equilibria for compartmental models of disease transmission. *Mathematical Biosciences*.

[34] Anderson RM, May RM (1991). *Infectious Diseases of Humans: Dynamics and Control*.

[35] Anderson RM, May RM (1982). *Population Biology of Infectious Diseases*.

[36] Sharomi O, Podder CN, Gumel AB, Song B (2008). Mathematical analysis of the transmission dynamics of HIV/TB coinfection in the presence of treatment. *Mathematical Biosciences and Engineering*.

[37] LaSalle JP (1976). *The Stability of Dynamical Systems*.

[38] Smith HL, Waltman P (1995). *The Theory of the Chemostat*.

[39] Iman RL, Conover WJ (1980). Small sample sensitivity analysis techniques for computer models, with an application to risk assessment. *Communications in Statistics A: Theory and Methods*.

[40] Iman RL, Helton JC, Campbell JE (1981). An approach to sensitivity analysis of computer models: part I—introduction, input variables election and preliminary variable assessment. *Journal of Quality Technology*.

[41] Iman RL, Helton JC (1988). An investigation of uncertainty and sensitivity analysis techniques for computer models. *Risk Analysis*.

[42] Blower SM, Dowlatabadi H (1994). Sensitivity and uncertainty analysis of complex models of disease transmission: an HIV model, as an example. *International Statistical Review*.

[43] McLeod RG, Brewster JF, Gumel AB, Slonowsky DA (2006). Sensitivity and uncertainty analyses for a sars model with time-varying inputs and outputs. *Mathematical Biosciences and Engineering*.

[44] Lakshmikantham V, Leela S, Martynyuk AA (1989). *Stability Analysis of Nonlinear Systems*.

